# CRISPR/Cas9-Based Genome Editing: Understanding Differences in DNA Repair Pathways, Profiles, and Outcomes

**DOI:** 10.3390/ijms27135905

**Published:** 2026-06-30

**Authors:** Samuel N. Effah, Shirley C. Barrera, Nahia Urturi Ortiz, Will Dampier, Michael R. Nonnemacher, Brian Wigdahl

**Affiliations:** 1 Department of Microbiology and Immunology, Drexel University College of Medicine, Philadelphia, PA 19102, USA; sne34@drexel.edu (S.N.E.); sb4476@drexel.edu (S.C.B.); nu62@drexel.edu (N.U.O.); wnd22@drexel.edu (W.D.); mrn25@drexel.edu (M.R.N.); 2 Center for Molecular Virology and Gene Therapy, Institute for Molecular Medicine and Infectious Disease, Drexel University College of Medicine, Philadelphia, PA 19102, USA; 3 Sidney Kimmel Cancer Center, Thomas Jefferson University, Philadelphia, PA 19107, USA

**Keywords:** CRISPR/Cas9, repair edit, repair profile, repair outcome, indel, target sequence, C-NHEJ, MMEJ, chromatin, prediction and analytical tools, HIV-1

## Abstract

Over a decade of advances in Clustered Regularly Interspersed Short Palindromic Repeats (CRISPR) and CRISPR-associated protein 9 (Cas9)-based technologies have culminated in the first-ever FDA-approved CRISPR/Cas-based therapy. Aside from this approved therapy for sickle cell anemia, several CRISPR/Cas-based therapies are currently under development or testing for a range of chronic diseases, including viral diseases like human immunodeficiency virus type 1 (HIV-1) infection, genetic diseases like familial hypercholesterolemia, and cancer. The success of these therapies hinges on the effective delivery of CRISPR/Cas9 components to target regions, efficient Cas endonuclease editing, repair profiles generated, and their resulting outcomes. Here, we discuss the factors that influence the generation of CRISPR/Cas9-generated repair edits, the overall profiles, and outcome prediction(s), as well as the analytical tools that have been developed to date. Finally, how this technology has been used towards a functional HIV-1 cure is discussed.

## 1. Introduction

Gene therapy is a fast-evolving and multifaceted medical field that includes, but is not limited to, correcting defective genes or knocking out disease-causing genes. Before the discovery of CRISPR/Cas9, gene editing tools like Transcription activator-like effector nucleases (TALENs) and zinc finger nucleases (ZFNs) were frequently used for genetic manipulation [1,2,3,4,5]. ZFNs are made up of a zinc finger DNA-binding domain and a restriction endonuclease that binds specific target DNA sequences and cleaves the DNA at the target site, respectively. TALENs possess a DNA-binding and restriction domain meant for DNA binding and cleavage, respectively. Though these tools exhibit efficient gene editing, they have some limitations [1,2]. Both tools require the engineering of new proteins to target different DNA sites [1,2,5]. This makes these tools less scalable, laborious, and expensive to use [1]. The uncovering of an adaptive immune system component of *Streptococcus pyogenes* (SpCas9) against bacteriophages, named Clustered Regularly Interspersed Short Palindromic Repeats (CRISPR) and CRISPR-associated protein 9 (Cas9) in 2012, has transformed gene editing capabilities and reach [6].

CRISPR/Cas9’s main attributes address the limitations of these earlier gene editing tools [3] through components such as an easily programmable single guide RNA (sgRNA) for different target sequences and the Cas9 protein that does not require re-engineering for every target. The Cas9 protein belongs to a Cas protein system, which is divided into classes, namely Class I (includes types I, III, and IV) and Class II (includes types II, V, and VI) [2,7,8,9,10]. Class II (of which the Cas9 protein belongs to) requires one Cas protein for its gene editing functions [1,7,10]. This feature makes its use simple, and they are well-studied and extensively used compared to the Class I systems, which require multiple Cas proteins for their function [11]. Other Cas proteins in the class II CRISPR system include Cas12 and Cas13 [12,13]. Cas12’s molecular targets include both dsDNA and ssDNA. Cas12 is smaller in size compared to SpCas9. Cas 12 also only requires crRNAs, targets Thymine-rich PAM sites, tends to have fewer off-target effects, and is effective with respect to inhibiting HIV-1 infections [12,13,14,15]. Cas13 targets RNA and does not induce double-stranded breaks (DSBs) reducing the chances of off-target effects associated with DNA double-stranded break (DSB) induction. It is also smaller compared to SpCas9, offers faster downregulation of genes, and is effective at inhibiting HIV-1 infections [12,13,15,16]. Despite the advantages of these Cas molecules, our review focused on SpCas9 and SaCas9 (*Staphylococcus aureus* Cas9) studies because these Cas9 proteins have been extensively validated with newer versions of these proteins providing more efficiency with less off-target effects [17]. The Cas9 proteins have a comprehensive toolkit of validated guide RNAs (especially SpCas9), and they are the most advanced in development with examples like their use in the recently approved sickle therapy (Casgevy) and in the investigational HIV-1 therapy EBT-101, currently in clinical trials (NCT05144386) [18,19].

The Cas9 protein consists of the guide RNA (gRNA) binding domains (REC1 and REC2 domains), the nuclease domains (RuvC and HNH domains), and the Protospacer Adjacent Motif (PAM) interacting domain [2,8,9]. The sgRNA consists of two components, namely the CRISPR RNA (crRNA) and trans-activating CRISPR RNA (tracrRNA) [1,3,10,20]. The crRNA is an easily modifiable oligonucleotide, usually 18–20 base pairs (bp) long, that directs the Cas protein to the target DNA site by hybridizing through complementation with the target sequence [2,3,21,22]. The tracrRNA is also modifiable, has defined secondary structures, and serves as a scaffold for the binding of Cas protein [2,10]. The Cas9 protein searches the genome for its PAM site while the sgRNA looks for complementarity with the stretch of nucleotides adjacent to the PAM site [1,3]. Next, the Cas9 protein will then create double-stranded breaks at least 3 bp from the PAM site [1]. CRISPR/Cas9 has been applied in diverse fields of medicine. CASGEVY, the first ever approved CRISPR/Cas9-based therapy, is a treatment for sickle cell disease and transfusion-dependent beta thalassemia [19,23]. Other therapies based on CRISPR/Cas9 currently in clinical trials include treatments for cancers like B-cell malignancies (NCT04637763), cardiovascular diseases like familial hypercholesterolemia (NCT05398029), autoimmune diseases like systemic lupus erythematosus (NCT06925542), inherited blood disorders like hemophilia B (NCT06379789), muscular dystrophies (NCT06594094), and infectious diseases like human immunodeficiency virus type 1 (HIV-1) infection (NCT05144386).

Despite these advances, there are hurdles associated with these CRISPR/Cas9-based therapies. The primary challenge is safety. Off-targeting and humoral/cellular immune response against CRISPR components and/or viral vectors used for delivery of these CRISPR components highlight these safety concerns [2]. These concerns have been extensively studied, leading to the implementation of several strategies, like sgRNA optimization, Cas9 protein modification, and low immunogenic viral vectors to minimize off-target effects and reduce host immune activation [2]. Another major hurdle for CRISPR/Cas9-based therapies is the effective delivery of CRISPR/Cas9 components into cells and tissues of interest [24,25,26]. In infectious diseases like HIV-1, CRISPR/Cas9 delivery efficiency is critical in preventing the chances of viral rebound [24,27,28]. Low immunogenic viral vectors (like the adeno-associated virus serotype 9 vectors), smaller Cas9 proteins (from *Staphylococcus aureus*), and nanoparticles are some of the strategies implemented to improve delivery efficiency [24,27,28]. However, these strategies at best have led to variable tissue/cell penetration [24]. The outcome of CRISPR/Cas9-mediated gene editing is also important in assessing the success of CRISPR/Cas9-based therapies.

**Figure 1:**
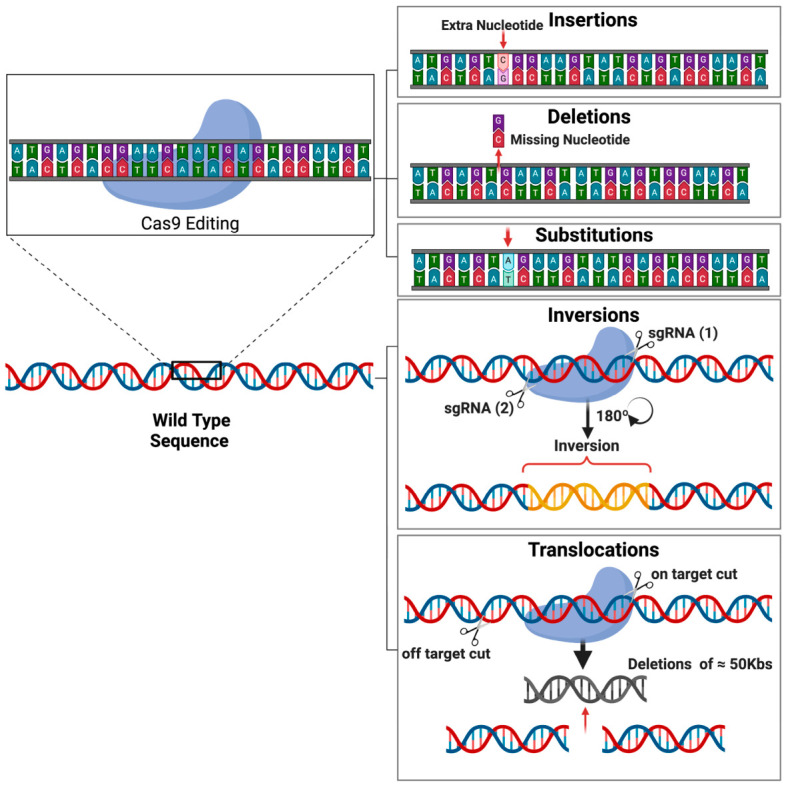
Types of CRISPR/Cas9-generated repair edits after DNA DSB Repair. DNA Repair edits generated after DNA DSB repair include indels (insertions or deletions) and other byproducts of DNA DSB repair, like substitutions, inversions, and translocations. **Insertion:** One or more nucleotide (nt) base pairs are added to the target DNA sequence. **Deletion:** One or more base pairs are deleted from the target DNA sequence. **Substitution:** One or more base pairs in the target DNA sequence are replaced with another nucleotide. **Inversion:** Occurs when a gene segment is cut off after dgRNA CRISPR/Cas9 editing, then annealed back in the reverse orientation. **Translocation:** Refers to deletions of more than 500 kb spanning an on-target break site to an off-target break site. Wild-type sequence refers to the original DNA sequence before CRISPR/Cas9 editing. Created in BioRender. Dampier, W. (2026) https://BioRender.com/tns1l6u.

We distinguish in this review between repair edit, repair profile, and repair outcome. We refer to repair edit as the individual double-stranded break repair products, like indels (insertion or deletion), or other complex repair byproducts like substitution, inversion or translocation observed after CRISPR/Cas9 editing (Figure 1; Table 1). Repair profiles refer to the frequency of the different repair edits in CRISPR/Cas9 edited cells (Table 1). Repair outcomes refer to the effect of these repair edits in CRISPR/Cas9 edited cells (Table 1). These effects of repair edits are either out-of-frame or in-frame mutations that may or may not alter the reading frame of the gene of interest. These repair edits and their outcomes determine the success of genetic editing in cells/tissues of interest. In this review, we focus on (1) repair edits, profiles and outcomes shaping gene knockouts, (2) factors that influence the generation of insertions or deletions, types of repair edits, and (3) repair profiles and outcome prediction and analytical computational tools, and then we (4) discuss the recent CRISPR/Cas9-based strategies paving the way towards an HIV-1 cure.

## 2. DNA DSB Repair Pathways, Repair Profiles and Outcomes

The complex of the endonuclease Cas9 enzyme and gRNA(s) creates DNA double-stranded breaks (DSBs) that are repaired by the host’s DNA DSB repair mechanisms [29,34,38]. The DSB repair process during V(D)J and class switch recombination (CSR) reactions in B lymphocytes has shaped the understanding of DSB repair mechanisms [45,46]. There is a general consensus on the two major repair pathways, the canonical non-homologous end-joining (C-NHEJ) repair pathway and the error-free homologous recombination (HR) or homology-directed repair (HDR) [45,46,47,48,49,50,51,52]. However, evidence for an additional pathway, the alternative end-joining (alt-EJ) emerged in subsequent years in various cellular systems including human, rodent, and yeast cells [45,47,53,54,55,56,57,58,59,60]. The nature of the alt-EJ repair process is still highly contested with some in the field suggesting the alt-EJ may not be a standalone repair process but a set of alternative components that could compensate for the C-NHEJ main proteins albeit with lower kinetic efficiency [46,48,61,62]. Though we recognize this opinion, we think this debate remains unsettled and therefore in this review, we maintain and discuss the role of the alt-EJ repair process in CRISPR editing but define the context in which we use this term (discussed below).

The C-NHEJ repair pathway is said to be active throughout the cell cycle, has faster repair kinetics, and is inherently precise [63,64,65]. It is a Ku (Ku70/Ku80)/DNA-PKcs/ligase 4 (Lig4)/x-ray cross complementation 4 (XRCC4)-dependent repair pathway with other factors like Artemis, XRCC4-like factor (XLF), polymerases (Pol) μ and λ, and the paralogue of XRCC4 and XLF (PAXX) also active in the pathway [47,48,50,54,66,67,68]. The Ku heterodimer proteins of Ku70/Ku80 have a high affinity for DNA DSB ends and prevent resection at the DNA break ends initiating the C-NHEJ pathway [54]. Ku proteins are DNA binding components of DNA protein kinase (DNA-PK) of which DNA-protein kinase catalytic subunit (DNA-PKcs) is a component [45,50,53,55,69,70]. Together, they serve as a scaffold for the recruitment of other C-NHEJ factors like Lig4 and XRCC4 [54,60]. DNA-PKcs is the main kinase responsible for phosphorylating itself and proteins in this pathway like Artemis, while Lig4 and XRCC4 form part of a ligation complex that ligates the DNA DSB terminal ends [45,48]. Lig4 and XRCC4 have no function outside C-NHEJ repair, and inhibition of these factors leads to a very severe form of C-NHEJ deficiency [56,66]. When the DSB ends requires further processing before ligation, nucleases like Artemis and polymerases like Pol λ or μ act on the ends [48,71]. Therefore, due to the incidence of DNA DSB ends that may not be able to be ligated and will require processing, repair by the C-NHEJ can also lead to indel formation aside from its intrinsic accurate repair [8,64,72]. These indels include small base-pair insertions, microhomology-dependent (1–4 bp), or independent small base-pair deletions [48,60,61,66,73].

C-NHEJ repair’s precise nature has been demonstrated in the repair of readily ligatable DNA blunt ends and in specific DNA DSB repair situations [64,74]. Some of these situations include DNA DSB repair involving the use of nascent RNA as a template for missing sequences, DNA DSB repair in human embryonic stem cells (hESCs) in the S/G2phase involving slower C-NHEJ kinetics and completion of a meiotic DNA DSB repair by C-NHEJ after initiation by HR [72,75,76]. The C-NHEJ’s mutagenic outcomes are of interest especially in therapies involving gene knockouts like the recently approved gene therapy for sickle cell disease (Casgevy) and for investigational therapies like CRISPR/Cas9-mediated inactivation of the HIV-1 provirus in HIV-1-infected cells (NCT05144386) [19].

Aside from the C-NHEJ repair, a slower and error-prone repair process associated with genomic instability and chromosome translocations and known as the alt-EJ repair has been described in C-NHEJ-deficient cells [50,53,54,66,70,77,78,79,80,81,82]. Early evidence for the activity of the alt-EJ repair process on endogenous chromosomal DSBs was seen in CSR reactions in B-cells deficient in Lig4 or XRCC4 and in Ku or Ku/Lig4 double-deficient B-cell CSR reactions [56,57,60,83,84]. Studies in different organisms support the unique activity of an alternative repair pathway which can even be active when HR and C-NHEJ repair pathways are intact [47,53,55,83,85]. The Ku/Lig4 independent form of alt-EJ strengthened support for the alt-EJ repair process as a standalone DNA DSB repair pathway [57]. This form of alt-EJ often uses microhomologies but is also involved in direct joining repair [57]. This has led some to describe it as a microhomology-mediated end-joining (MMEJ) repair; however, not all alt-EJ repair is MMEJ [86,87,88,89,90].

Another form of alt-EJ repair, described as a Ku-dependent, but Lig4 or XRCC4 independent, relies on longer microhomologies (MHs) and uses either ligase 1 (Lig1) or ligase 3 (Lig3) has also been proposed [57]. However, there is evidence that the MMEJ repair pathway is dependent on Lig3 [47,48,83,87] and that a second alt-EJ mechanism known as alt-NHEJ is reliant on Lig1 [83,87,91]. Lig1 has also been proposed as a backup to Lig3 [83,87]. Conversely, in Lig3-deficient B cells, the extent of microhomology use in CSR reactions was not impacted, leading to the proposal that there may be redundancies between Lig 1 and 3 usage [45,47,48,80,92]. Whether Ku-dependent or not, a defining attribute of the alt-EJ repair is its dependence on pre-existing or generated microhomologies (2–20 bp in length) [56,57,88,93].

Microhomology use is not exclusive to alt-EJ but is also involved in some C-NHEJ repair [47,48,56,59,73,83,94]. Therefore, microhomology (MH)-mediated deletions in alt-EJ repair are thought to more often involve microhomologies of >5 bp distinguishing it from MH-mediated deletions associated with C-NHEJ repair [47,48,90]. Some of the factors associated with MMEJ or alt-EJ repair aside from Lig3, include Poly ADP-ribose polymerase 1 (PARP1), polymerase theta (Polθ), meiotic recombination 11 (MRE11), X-ray repair cross-complementing protein 1, (XRCC1), and carboxy-terminal binding protein (CtBP)-interacting protein (CtIP) [47,50,52,79,80,83,84,90,95,96,97,98]. Inhibition of these factors has been shown to reduce MH junctions in CSR reactions [47] and large base-pair deletions (often MH-based) in CRISPR editing experiments [39]. Again, in cancer studies, inhibition of PARP1 proteins and other MMEJ-associated factors has been shown to enhance synthetic lethality in HR-deficient cells [47,83,93,99,100]. Though these factors have been associated with the alt-EJ (or MMEJ) repair pathway, they are also known to have overlapping roles in other DNA repair pathways questioning their exclusivity to the alt-EJ repair pathway [45,47,50]. Based on this evidence, we describe in this review the C-NHEJ repair as Ku/DNA-PKcs/Lig4/XRCC4 dependent and focus on the MMEJ repair (a type of the alt-EJ repair) describing it as Ku/Lig4 independent but facilitated by Lig3, PARP1 and Polθ.

In CRISPR gene knockouts, the C-NHEJ and MMEJ repair mechanisms play major roles in shaping repair profiles [32,38,101]. Inhibition of the C-NHEJ pathway has also been shown to lead to MMEJ repair of DNA DSBs [4,65]. Repair profiles generated by these DNA repair pathways can result in frameshift mutations that alter the reading frame of protein domains leading to gene knockout [30,41]. In this section, we review the frequency of repair edits (repair profiles) generated by these DNA DSB repair pathways in in vitro knockout studies and discuss the outcomes of these repair edits.

### 2.1. Repair Profiles

Insertions and deletions, commonly referred to as indels, are the most predominant repair edits generated after CRISPR/Cas9 mutagenesis (Figure 1; Table 1) [29,30,32,34]. However, several studies report other types of repair edits, including complex combinations of insertions and deletions, nucleotide substitutions, inversions, and translocations (Figure 1; Table 1) [32,35,36,44,102]. For sgRNA CRISPR editing without template DNA, deletions are generally observed more frequently than insertions [30,32,34,36,37]. This observation is subject to factors like the target sequence [30,101], time [65,101], DNA DSB repair pathway [39], and chromatin environment [103]. Small base-pair (1–2 bp) insertions and deletions are also some of the most frequent indel classes (Table 1) [29,30,33,101]. High frequencies of these small base-pair indels are usually observed at early time points between 4 and 48 h (depending on the delivery method of the CRISPR components) after CRISPR/Cas9 editing [4,30]. Larger deletions and insertions are also observed at later time points (≥48 h) [4,101]. The frequencies of both small and larger deletions are primarily target sequence-dependent. However, the frequencies of these larger deletions are also associated with the existence of microhomologies in the target DNA sequence [4,101]. CRISPR editing in stem cells (both in humans and mice) has also been shown to preferentially lead to the selection of larger insertions and deletions compared to other cell lines [30]. Unedited, uncut, or wild-type sequences also persist after CRISPR/Cas9 delivery, and the frequencies of these vary among studies and are likely influenced by the target sequence [35,37,40].

Nucleotide substitutions, one of the less frequent repair edits, have a lot of skepticism surrounding their identification and quantification [37,44,102]. In some studies, they are identified but removed from analysis because very few tools can validate their presence [29,32,37]. This is because these nucleotide substitutions have also been associated with sequencing or DNA polymerase errors [37]. However, CRISPR analysis tools like CRISPR-Sub have been designed to distinguish CRISPR-induced substitutions from false-positive substitutions associated with DNA polymerase errors (Table 1) [37]. Analysis of unedited or wild-type sequences has been shown to harbor these nucleotide substitutions after comparisons with the mock edited sequences [37]. These findings underscore the need to develop more tools that accurately identify nucleotide substitutions in CRISPR-edited sequences. Another low-frequency repair edit called translocations refers to deletions usually >500 kb that span either an on-target break site and an off-target break site within a chromosome (intra-chromosomal) or an on-target break site and a genome-wide break site (inter-chromosomal) (Figure 1; Table 1) [11,35,36,104]. These translocations have been associated with chromosomal instability and oncogenesis, a potential drawback for CRISPR editing [36,104].

The number of gRNAs used in CRISPR editing can also impact repair profiles [40,41]. Dual-guide RNA (dgRNA) utilization in template-free CRISPR editing has led to higher gene knockout efficiency compared to sgRNA use (Table 1) [40,43]. This limitation of sgRNAs is significant and has been demonstrated especially in viral gene knockout studies (e.g., HIV-1, HSV-1, and HCMV) where viral escape mutants have been reported after sgRNA CRISPR editing [43,44,105]. Viral escape mutants usually have mutations at the target site where gRNAs bind, thereby preventing hybridization between the gRNA and viral target DNA sequence, subsequently nullifying the Cas9 endonuclease activity [43,44,105]. Outside viral gene knockout studies, some sgRNA CRISPR studies have shown that certain genomic regions are more prone to in-frame mutations, which do not disrupt the reading frame of human genes, reducing knockout efficiency [33,34].

These drawbacks have necessitated a dual- or multiplex-gRNA approach, utilizing two or more gRNAs. Studies implementing a dgRNA approach reveal that two of the most frequent repair edits are deletions spanning the target cut site of both gRNAs after simultaneous Cas9 editing (excision events) and mutation(s) either at one cut site or at both (hypermutations) [40,43,44]. Hypermutations at either one cut site or both may include deletions, insertions, or substitutions. Other lower-frequency repair edits after dgRNA CRISPR editing include inversions and a complex combination of excision events and insertions [40,42,43]. Inversion events occur when excised DNA segments after dgRNA CRISPR editing undergo a 180° flip and are re-ligated back or are re-inserted in the reverse orientation (Figure 1; Table 1) [43,106]. Again, several of these studies involving a dual/multiple gRNA approach report lower frequencies of unmutated/wild-type sequences compared to the sgRNA approach [40,44]. A study involving a three-gRNA approach also showed the presence of excision events between the first and second gRNA target cut site and the second and third gRNA target cut site as well as an excision event spanning the first and third gRNA target cut site [41]. In summary, these hypermutation and excision events have demonstrated enhanced gene inactivation, making a multiplex-gRNA approach a valuable tool for studies where gene knockout efficiency must reproducibly remain high for success [40,43,44]. An example of this is in viral gene knockout studies in HIV-1, HCMV, and HSV-1, where reproducibly high viral gene knockout efficiency is essential in propelling dgRNA CRISPR approaches as potential therapies, and more importantly, preventing viral escape mutants [43,44,105].

### 2.2. Repair Outcomes

**Figure 2:**
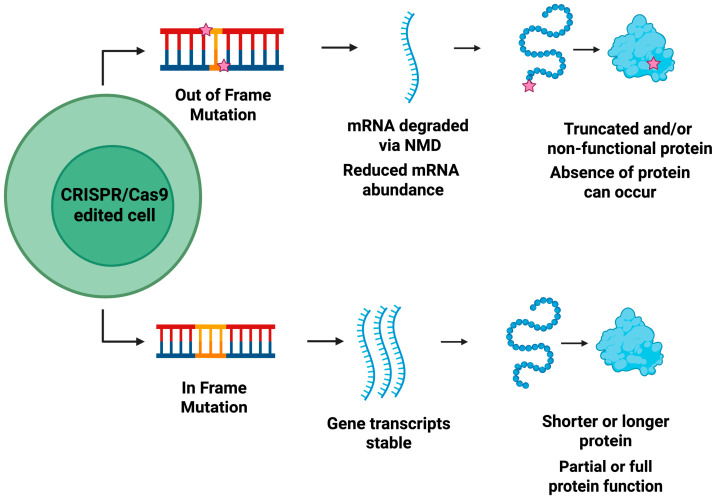
Types of DNA repair outcomes derived from DNA repair edits after DNA DSB Repair. DNA repair edits (insertions, deletions, substitutions, inversions, and translocations) can either lead to in-frame or out-of-frame mutations (repair outcomes). Out-of-frame mutations can lead to nonsense-mediated mRNA decay (NMD) due to the presence of premature termination codons (PTC) and subsequently reduced mRNA abundance. In-frame mutations often lead to stable gene transcripts; however, they may create shorter or longer proteins. The change in the length of the protein may lead to partial protein function or no effect on protein function. Created in BioRender. Dampier, W. (2026) https://BioRender.com/85kdvjo.

CRISPR/Cas9 editing can lead to in-frame and out-of-frame mutations, resulting in either the maintenance of the coding region of genes, albeit shorter or less functional proteins, or a complete disruption of the reading frame with the introduction of a premature stop or termination codon, respectively (Figure 2) [29,30,39,40,107]. In-frame mutations are regarded as deletions or insertions that are 3 bp or multiples of 3 bp long that do not disrupt the reading frame of genes, given that three nucleotide base pairs comprise a protein codon [30,105]. In-frame mutations generated under CRISPR/Cas9 gene knockout experiments can reduce gene knockout efficiency. This could mean residual disease-associated protein production from disease-causing genes or in the case of HIV-1 therapeutics, persistence of infected cell populations with an active provirus or production of HIV-1 escape mutants [43]. Some strategies developed to reduce the frequency of these in-frame mutations have employed the use of dual gRNAs instead of a single-guide RNA [40,41,43,44]. These dual-gRNA approaches have been shown to increase deletion frequency, especially deletion of the intervening sequence, increasing the possibility of out-of-frame mutations that increase knockout efficiency [40,41,43,44].

The frequency of these in and out-of-frame mutations varies amongst studies, and they are dependent on factors such as the length of indels, the target sequence, and the number of gRNAs targeting a specific site (Table 1) [30,40,44]. Several studies show a positive correlation between an increase in knockout (KO) efficiency (a result of frameshift mutations) and an increase in sequence reads containing indels (Table 1) [40,41]. Higher frequencies of out-of-frame mutations leading to gene disruptions and greater KO efficiency have been associated with dgRNAs compared to sgRNA use [40]. In silico tools such as FORECasT, InDelphi, and SPROUT leverage machine learning algorithms and predict the likelihood of in-frame and out-of-frame mutations (Table 2). Findings generated by these tools show a high positive correlation between predicted and observed in or out-of-frame mutations (Table 1) [30,31,34,38]. In addition to predicting indel classes (insertions and deletions) and their frequencies, some of these prediction tools can also identify in-frame mutations in silico. This is valuable in showing how, for example, in-frame mutations can restore the reading frame of disease alleles with frameshift mutations (e.g., in Duchenne muscular dystrophy), thereby recovering protein production to a biologically significant extent [38,42]. Using tools like inDelphi, precision gRNAs have been designed that facilitate precise template-free CRISPR editing (Table 1) [34]. inDelphi provides a functionality that facilitates the identification of high-precision gRNAs (Table 1). inDelphi considers gRNAs with at least 30 bp of sequence upstream and downstream of the cleavage site and provides precision of gRNA as a statistical measure of the frequency of distribution of indels, with high precision gRNAs containing a limited number of repair outcomes accounting for most outcomes [34]. These precision gRNAs have been used to predictably generate specific indels, like 1-base pair insertions in endogenous relevant human disease alleles [34]. The precision editing of some of these precise gRNAs has also been enhanced to generate precise microhomology-mediated deletions in genes with pathogenic microduplications through inhibition of C-NHEJ-associated proteins [34].

However, these approaches certainly have some limitations since they may not completely restore the protein levels to wild-type levels to completely revert the disease condition [34,42]. Therefore, there needs to be a case-by-case evaluation of these diseases causing frameshift mutations to assess whether the introduction of in-frame mutations provides biologically relevant and significant correction to the reading frame of these disease alleles [34,42]. Alternatively, depending on the characteristics of the disease-causing frameshift mutation, homology-directed repair-guided CRISPR editing can be employed [29,32].

### 2.3. CRISPR/Cas9 Delivery Approaches

CRISPR/Cas9 approaches (in vitro), like the delivery of CRISPR/Cas9 components into cells, can also impact Cas9 activity and repair profiles (Table 1) [29,31,41,44]. Some studies report that stable integration of CRISPR/Cas9 components (e.g., through CRISPR lentivirus transduction) into the genomes of cells leads to mature editing profiles at later time points compared to the transient introduction of these components (e.g., through lipofection of CRISPR/Cas9 plasmids or nucleofection/electroporation of CRISPR ribonucleoprotein complex(es) (RNPs) [41,44]. Early timepoint indel profiles are associated with the C-NHEJ repair and can be prone to re-cleavage at later time points, favoring large base-pair deletions [101]. These large base-pair deletions have been associated with MMEJ repair [101]. Mature repair profiles do not change much in the presence of Cas9 and gRNA activity due to the accumulation of mutations in the target DNA sequence after repeated re-editing of repaired CRISPR/Cas9-mediated cuts [41,101]. This prevents the hybridization between the gRNA and the target site and subsequently Cas9 activity. Mature repair profiles, which occur at later time points (5–14 days), include indel classes such as larger base pair deletions compared to single base-pair deletions and insertions at early time points (4–48 h) [41,44,101].

Constitutive expression of Cas9 endonuclease enzyme via delivery methods like lentivirus transduction ensures persistent Cas9 activity compared to transient methods like electroporation and lipofection. Though constitutive delivery of CRISPR components requires additional steps to deliver Cas9 RNP to the nucleus compared to transient methods like electroporation, a study by Karp et al. showed that constitutive delivery of Cas9 RNPs ensured faster editing kinetics compared to electroporation [112]. Transient introduction of CRISPR components, like in the electroporation of cells, can lead to cellular degradation of these components within 72 h, thereby stalling the maturation of early repair profiles [40,41]. Transient delivery of plasmids encoding CRISPR components into cells has also been shown to have delayed Cas9 activity compared to CRISPR RNPs [113]. Regardless of the delivery method, a study by van Overbeek et al. also showed that the indel profiles or classes themselves remained relatively similar though the relative frequency of these indel profiles or classes can be different [101].

Some studies also show that the cell type can play a role in the class of indels that are generated after CRISPR editing [30]. Interestingly, CRISPR editing in stem cells tends to produce a higher frequency of large base-pair deletions compared to somatic or immortalized cell lines [30,34,38].

## 3. Factors Influencing Indel Profiles

**Figure 3:**
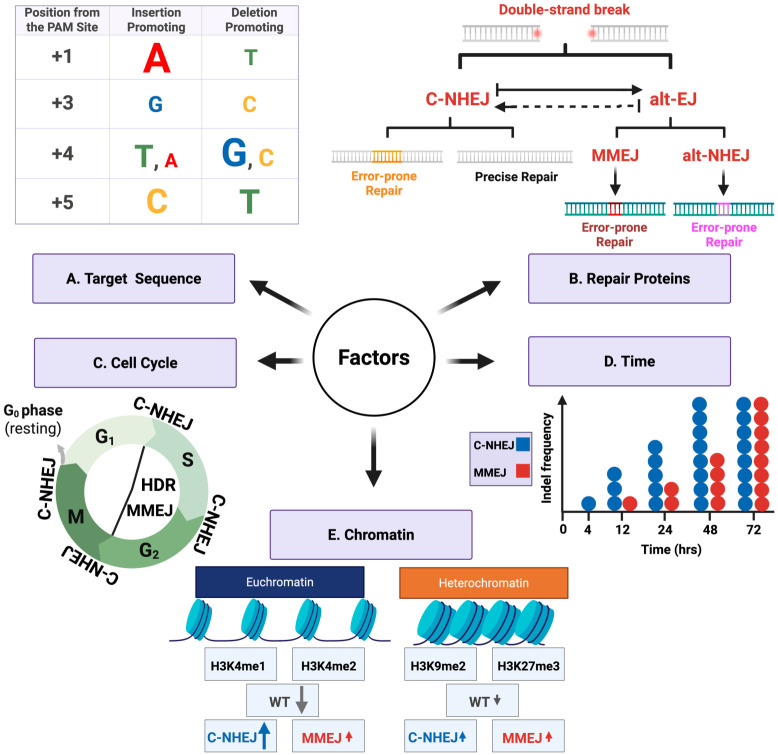
Factors influencing CRISPR/Cas9-induced indel profiles. CRISPR-generated indel profiles are shaped by multiple interconnected factors, including the target sequence, DNA repair proteins, cell cycle stage, time after editing, and the local chromatin environment. (**A**) **Target sequence:** The nucleotides flanking the Cas9 cut site, particularly at positions +1, +3, +4, and +5 relative to the PAM site, significantly influence the frequency and type of indels generated. In this representation, the larger the letter, the greater its influence is in promoting either an insertion or a deletion. (**B**) **DSB repair pathways and proteins:** Inhibition of the C-NHEJ pathway results in a reduction in small base-pair insertions and deletions, thereby promoting the MMEJ pathway (an alt-EJ repair process). In contrast, suppression of the MMEJ pathway shifts the repair of these DNA DSB cuts towards the generation of C-NHEJ-type indels. (**C**) **Cell cycle:** The C-NHEJ pathway operates throughout the cell cycle, while MMEJ and HDR are more active in S/G2 phases. (**D**) **Time:** The duration of Cas9 activity shapes the evolution of indel profiles. C-NHEJ (in blue)-associated small indels dominate early (within 12–24 h), whereas MMEJ (in red)-associated large deletions emerge later (24–48 h). After 72 h, larger deletions happen more frequently as well, a characteristic of the MMEJ. (**E**) **Local chromatin environment:** Euchromatin, with markers (in black) like H3K4me1 and H3K4me2, enhances Cas9 accessibility and editing efficiency, increasing indel frequency and favoring the C-NHEJ (in blue) pathway over the MMEJ (in red) for the DNA DSB repair. However, heterochromatin, with H3K9me3 and H3K27me3 markers, limits Cas9 access and shifts the DNA repair pathway balance, maintaining high levels of wild-type sequences and favoring low levels of both C-NHEJ (in blue) and MMEJ (in red) indels. Created in BioRender. Dampier, W. (2026) https://BioRender.com/75kud00.

CRISPR-generated indel profiles are mostly not random and reproducible [29,30,65,101,114]. Several factors have been implicated in influencing how these indel profiles are generated. These factors primarily include the target sequence, DNA DSB repair pathways and repair proteins, cell cycle, time (duration of Cas9 editing), and local chromatin environment (Figure 3) [30,39,65,101,103]. This section highlights the roles these factors play in CRISPR-generated mutagenesis.

### 3.1. Target Sequence

CRISPR-generated indel profiles are strikingly reproducible when the target DNA sequence is similar compared to when the target DNA sequence is different [30,101]. This has been shown in repeated experiments involving the same gRNA and target sequence [30,65,101]. In one such study, spacer sequences, which were described as similar repeated sequences across the genome (repeated 2–14 times) in mammalian cells, were shown to have similar indel profiles after CRISPR editing [101]. Though the relative indel frequencies differed depending on factors such as cell type, delivery, and editing efficiency, the top indel classes or indels with the highest frequencies remained similar when targeting the same target sequence [101]. In another example, though CRISPR editing efficiencies in stem cells and primary cells are known to be lower compared to mammalian cell lines, the CRISPR-generated top indel profiles were generally similar when the target sequence and gRNA were similar [34,101]. Again, though transient delivery of CRISPR components like the electroporation of CRISPR RNPs or stable delivery of CRISPR components through lentivirus transduction may lead to early and mature indel profiles respectively, and the top indel profiles are usually similar and target sequence dependent [101].

The nucleotides around the cut site of the target sequence are a major determinant of Cas9-induced indels [29,30,31,34]. The HNH domain of the Cas9 endonuclease cuts the complementary target DNA strand after gRNA: DNA hybridization, specifically three base pairs upstream of the PAM site. However, the RuvC domain of the Cas9 endonuclease can cut the non-complementary strand at multiple sites of three to six base pairs from the PAM site [8,29]. This flexibility of the Cas9 domains can either create blunt ends at the break site when both domains cut opposite DNA strands at the same position upstream of the PAM site or staggered ends when these Cas9 domains cut at different sites from the PAM site [8,29,115].

Most of the Cas9-induced cuts are blunt-ended, with the minority being staggered [114,115]. The most frequent staggered ends are 1–2 base pair 5′ overhangs [115]. The staggered cut ends can lead to the filling of the overhangs by DNA polymerase 4 leading to templated insertions, or the processing of the overhangs by exonucleases, leading to templated deletions at the cut site [29,30,114]. DNA Polymerase 4 is error-prone; therefore, it can incorporate random nucleotides leading to non-templated insertions [114]. The blunt ends frequently lead to precise C-NHEJ repair or can be processed by exonucleases to generate C-NHEJ-mediated indels [29]. For most templated insertions and deletions, the 4th nucleotide from the PAM site or -1 nucleotide from the cut site, where the cut site refers to 3 bp from the PAM site, plays an important role in determining which template insertions and deletions are generated (Figure 3A) [29,30,31,34,114]. The 3rd (+1 nucleotide from the cut site), 1st (+3 nucleotide from the cut site/adjacent PAM site), and 5th (-2 nucleotide from the cut site) nucleotides from the PAM site have also been shown to be involved in the determination of templated insertions and deletions (Figure 3A) [29,31,34].

The majority of +1 insertions are templated [32,114]. Both 1 and 2 bp templated insertions are dependent on the nucleotides at and around the DNA DSB cut site [32]. A thymine (T) nucleotide at position 4 (4th nucleotide from PAM site) in the target sequence has been shown to promote single-base insertions, while a guanine at the same position is known to promote single-base pair deletion (Figure 3A) [29,30,31,34]. Adenine (A) at position 4 also generates single-base pair insertions, though less frequently than Thymine at the same position [29,31,34]. A guanine at position 3 promotes the generation of single-base insertions, and a cytosine (C) at position 3 promotes single-base pair deletions (Figure 3A) [29,31,34]. At position 5, Cytosine promotes insertions while Thymine promotes deletions (Figure 3A) [31,34]. TG dinucleotide flanking the cleavage site is mostly biased towards the generation of insertions [32]. However, a GA dinucleotide flanking the break site usually leads to the generation of deletions [32]. Evidence shows that if Thymine is the 4th nucleotide from the PAM site, it is very likely that the inserted nucleotide after C-NHEJ repair is also Thymine [29,30]. The frequency of this assertion tends to decrease in this order if the nucleotide is an A, C or G. If there is a homopolymer dinucleotide (X|X) surrounding the cut site in a target sequence, one of the repeated nucleotides will likely be removed to generate a templated single base pair deletion (X) [30].

Most of the 2-base pair templated deletions arise from a heteropolymer nucleotide pair at each side of the DNA DSB cut site (XY|XY), with the resultant 2-base pair deletion being a removal of one of the heteropolymer nucleotide pairs at the cut site (XY) [30]. Amongst homopolymer multiple base nucleotide insertions (which are 2–3 bp insertions), many of them are multiple base insertions of Thymine (e.g., TT or TTT) [114]. The frequencies of other homopolymer multiple base pair nucleotide insertions decreased in the order of Adenine (AA or AAA), Guanine (GG or GGG), and Cytosine (CC or CCC) [114]. However, the majority of these homopolymer multiple base pair nucleotide insertions seem to be templated by the base that was inserted in the initial +1 base pair insertion [114]. These rules governing templated insertions and deletions are non-random and reproducible in several studies [29,31,32,34].

### 3.2. DNA DSB Repair Pathways and Proteins

CRISPR-mediated gene inactivation leads to indel profiles that have specific contributions from two major DNA repair pathways, namely the C-NHEJ and MMEJ (Figure 3B) [30,101,116]. The immediate-early and early responses after the induction of a DNA double-stranded break have been extensively reviewed in [63,117]. The choice of these DNA repair pathways depends on factors such as the duration of Cas9 editing [29,101], the availability of enzymes at the DNA DSB cut site [30,103], cell cycle stage [8,30,89,118], and the local chromatin environment [103,119,120,121], all discussed below. The level of DNA end resection also plays a major role in deciding which DNA repair pathway is employed to fix DNA DSB. After the DNA damage response (DDR) factor 53BP1 is recruited to the DNA DSB site, it inhibits BRCA1, suppresses DNA end resection and facilitates the C-NHEJ repair [117,122,123]. In the S/G2 phase, BRCA1 antagonizes 53BP1-mediated facilitation of C-NHEJ and promotes HR [124]. Factors like the Ku heterodimer protein complex protect the DNA DSB ends from resection initiating the C-NHEJ repair pathway. CtIP and the MRN complex (Mre11, Rad50, and Nbs1) binding lead to small DNA resection at the break site, facilitating either MMEJ or repair by HR [103,119].

The kinetics of activity between C-NHEJ and MMEJ repair also differ [29]. C-NHEJ repair occurs within hours of Cas9 editing, while MMEJ occurs as early as 16 to 24 h to about 2 to 3 days after Cas9 editing [4,29,65,103]. The C-NHEJ repair pathway has been associated with both precise and error-based repair. The error-based C-NHEJ repair leads to small base-pair insertions and deletions, while the precision-based C-NHEJ leads to restoring the edited target sequence to its wild-type sequence (unmutated) [121]. The MMEJ repair may occur at later editing time points, is dependent on the presence of microhomology sequences, contributes to the formation of varying subsets of deletions that occur after CRISPR editing, and is activated after a small level of resection at the DNA DSB site [4,8,30,38,125]. Factors that influence the type of MH-deletions include the distance between the microhomology sequences and the length of the microhomology sequence [30,36]. Therefore, this should mean greater frequencies of MH-deletions are observed when the distances between the long base-pair microhomologies are separated by short base-pair distances [30,36]. Interestingly, MH-deletions arising from small base-pair microhomologies are frequently found in mutated reads [29,126]. Chemical inhibition of proteins or gene knockout of genes in these DNA repair pathways also impacts indel profiles [101]. The inhibition of C-NHEJ-associated repair proteins like DNA-PKcs and Lig4, either through chemical perturbation or gene knockout, has been shown to reduce the frequencies of small base-pair (1–3 bp) insertions and deletions while favoring larger MH-deletions (Figure 3B) [30,39,101,125]. Conversely, the inhibition of MMEJ-associated proteins like Polθ. and Lig3 are known to reduce MH-deletions while increasing the frequencies of C-NHEJ-associated indels (Figure 3B) [30,39,125]. This is also evident in cell lines like HCT116, where large base-pair deletion frequencies are reduced due to the inhibition of the MMEJ repair pathways [101]. Taken together, DNA DSB repair proteins influence CRISPR-mediated indel profiles [30,39,125].

### 3.3. Cell Cycle

The extent of C-NHEJ and MMEJ usage by mammalian cells also depends in part on the cell cycle stage (Figure 3C) [8,30,89,118]. Key resection factors like MRN complex and CtIP recruited at specific cell cycle stages (S/G2 phase) can shift the choice of the DNA DSB repair towards the homology-directed repair (HDR) and MMEJ repair pathway and away from the C-NHEJ repair pathway [8,89]. The choice for HDR or MMEJ during the S phase is also dependent on the phosphorylation of CtIP and Sae2 by Cyclin-dependent kinases (CDKs) [127,128,129,130]. The level of resection in the MMEJ pathway is minimal compared to HDR (<20 base pairs), which requires extensive resection [89]. Generally, C-NHEJ can be utilized throughout the cell cycle by mammalian cells; however, HR is restricted to the S/G2 phase, where there are available template DNA/sister chromatids (Figure 3C) [8,89,118]. MMEJ activity is low during the G0/G1 phase, but it is predominantly employed in the S/G2 phase (Figure 3C) [8,89]. MMEJ does not require template DNA for its activity but rather requires the presence of microhomologies (5–20 base pairs) around the break site [8,89]. Therefore, the impact of the cell cycle on CRISPR-generated indels is through the regulation of DNA DSB repair pathways.

### 3.4. Time

The Cas9 endonuclease enzyme can stay bound to the cut site hours after its initial edit [30,39]. Again, constitutive expression of Cas9 in mammalian cells through delivery methods like lentivirus transduction provides the enzyme the opportunity to re-cleave repaired cut sites [30,39,44,101]. Therefore, the duration with which Cas9 interacts with its target site can influence the indels that are generated. Small base-pair deletions and insertions mostly dominate early timepoints CRISPR indels (≤48 h) (Figure 3D) [29,44,101]. However, larger deletions constitute a significant proportion of mature indel profiles or are comparable to the frequency of small base-pair deletions at later time points (Figure 3D) [29,44,101]. Interestingly, cell type has also been shown to be a determinant in the time taken to accumulate mature indel profiles [131]. It has been shown that the repair kinetics of post-mitotic cells like neurons are slower compared to rapidly dividing cells like human-induced pluripotent stem cells (iPSCs) [131].

Some larger deletions, associated with re-cleavage of repaired DNA DSB cuts [30,39], are also known to be generated by the MMEJ repair pathway (an error-prone end-joining DNA repair pathway known to be activated at later time points) [29,65]. Larger deletions or an increase in deletion frequencies could therefore alter the ratios between in-frame and out-of-frame mutations impacting knockout efficiencies. One base pair insertion is the dominant insertion class at both early and late time points [29]. At a lesser frequency, 2-base pair insertions are also present at both time points [29]. Larger insertions > two base pairs are rare at both time points in mammalian cell lines [29]. These observations put into context factors that will determine how we assess mature repair profiles and evaluate knockout efficiencies. Overall, the duration of Cas9 activity plays a role in shaping CRISPR-mediated indel profiles.

### 3.5. Local Chromatin Environment

The local chromatin environment surrounding the target sequence is known to influence both Cas9 binding and editing efficiency [33,119,121,132,133,134]. However, there seem to be a lot of nuances when it comes to the influence of the local chromatin environment on CRISPR/Cas9-mediated indel profiles [33,103,119,121,135]. Target sequences can be surrounded by diverse chromatin environments [33,119,136]. These include regions of heterochromatin, euchromatin and regions that are a mixture of heterochromatin and euchromatin [103,119,136]. Heterochromatin regions are subclassified into constitutive heterochromatin, facultative heterochromatin, heterochromatin associated with LAD (Lamina-associated domain) and markers like H3K9me2 or a combination of these three (Triple heterochromatin) (Figure 3E) [119,137]. Constitutive heterochromatin is a highly condensed region that may include regions of deacetylated histones and trimethylation of lysine 9 of histone 3 (H3K9me3) [119,136,137]. Facultative heterochromatin is a conditionally condensed region (more plastic) and can decompact under stimuli [137]. They can include regions of trimethylation of lysine 27 of histone 3 (H3K27me3) (Figure 3E) [119,136,137].

Euchromatin regions are associated with transcribed genes, gene enhancers, or regions characterized by H3K4me1, H3K4me2, and H3K27ac (Figure 3E) [103,119,121]. Heterochromatin regions surrounding the target sequence hinder Cas9 binding and activity, thereby decreasing CRISPR efficiency [103,119,121,132,135]. Euchromatin regions provide increased access to target sequences, which subsequently enhances Cas9 binding and CRISPR editing efficiency [103,119,132,135]. Heterochromatin regions have also been shown to decrease total indel frequencies, while euchromatin regions increase these indel frequencies [119]. Together, these observations highlight the influence of the local chromatin environment on CRISPR editing efficiencies [33,119].

There are nuanced opinions when it comes to the impact of chromatin on the CRISPR-mediated indel profiles [33,103,121,132,135]. Evidence from studies opposing chromatin impact on CRISPR-mediated indel profiles shows that though chromatin environment impacts Cas9 binding and editing, there is no change in the top indel classes after CRISPR/Cas9 editing, although there may be some noticeable changes in their relative frequencies [33,132,135]. However, other studies suggest that the local chromatin environment may have a subtle impact on CRISPR-mediated indel profiles, and this is likely through the local chromatin environment’s impact on DNA DSB repair pathways [33,103,121]. This influence on the DNA DSB repair pathway by the local chromatin environment can subsequently lead to changes in both indel profiles and their relative frequencies [103,121]. Studies show that C-NHEJ-mediated factors like Lig4, DDR factors like 53BP1, MMEJ-mediated factors like Polθ and CtIP and HDR-mediated factors like RAD51 and MRE11 are detected at the break site regardless of the chromatin environment [103]. Though these proteins bind at the break site irrespective of the chromatin environment, immunoprecipitation studies show differential binding or avidity at the break site that is chromatin-dependent [103,120,121,138].

These DNA DSB proteins can also influence the MMEJ: C-NHEJ ratio at the cut site [103]. Unsurprisingly, factors like Lig4 correlate negatively with the MMEJ: C-NHEJ ratio, while Polθ correlates positively with this ratio [103]. A higher MMEJ: C-NHEJ ratio enhances MMEJ activity and correlates with heterochromatin regions, while a lower MMEJ: C-NHEJ ratio correlates with euchromatin regions (Figure 3E) [8,103,120]. It must be stated that the enhancement of MMEJ in heterochromatin regions is also dependent on the type of heterochromatin region and C-NHEJ still plays a role in repairing CRISPR-mediated cuts in heterochromatin as well as euchromatin regions (Figure 3E) [103]. The type of target sequence in conjunction with the local chromatin environment may also impact the indel profiles generated [33]. In one such study, the impact of chromatin on indel classes was investigated using precise and imprecise targets [33]. Imprecise targets were defined as target sequences that generate low-frequency top indel classes, while precise targets are sequences that generate top indel classes with high frequency [33]. Precise targets also had, on average, more indels compared to imprecise targets after CRISPR editing, and lastly, precise targets showed a strong bias towards single-nucleotide indels, while indels generated from imprecise targets varied widely [33]. The features of imprecise sequence targets make them more susceptible to chromatin-modulating drugs like TSA, thereby influencing changes in both the CRISPR-mediated top indel classes and their frequency [33]. However, in precise targets, the top indel classes remained the same [33]. Together, these studies show that the target sequence remains a major determinant of CRISPR-induced indels, but the local chromatin environment may influence a given target’s site top indel classes and their frequencies.

## 4. Computational Tools for Repair Profile and Outcome

### 4.1. Prediction Tools

To understand CRISPR/Cas9 repair-mediated profiles and outcomes, including their genomic distribution, computational tools have been developed for both upstream in silico prediction and downstream experimental analysis [29,30,31,32,34,35,36,37,40,51,110,111,139,140,141,142,143,144,145]. The development of advanced computational prediction tools has enhanced the ability to anticipate CRISPR/Cas editing outcomes, equipping investigators with powerful approaches to predict the results of CRISPR/Cas gene editing and guide experimental design prior to experimentation [30,31,32,34,110,111]. This is useful in pre-selecting a gRNA with a desired repair product, identifying probability and/or frequency of intended or unintended editing events, leading to the assessment of potential risks and benefits within the experimental design. Key features relevant to tool utilization include their configuration and gene editing outcome prediction (Table 2). Among the CRISPR/Cas editing prediction tools identified in this review, six integrate a machine learning model in their configuration: (1) inDelphi utilizes a neural network and multitask framework learning; (2) Favored Outcomes of Repair Events at Cas9 targets (FORECasT)—a multi-class logistic regression model; (3) Logistic Regression Model to Predict Insertions and Deletions (Lindel)—a logistic regression model; (4) CRISPR Repair Outcome (SPROUT)—gradient boosted tree/decision trees; (5) Apindel—a GloVe model along with Bidirectional Long Short-Term Memory (BiLSTM) network, Attention mechanism, Positional Encoding; (6) CRISPR Outcomes Through cONvolutional neural networks (CROTON)—a Convolutional Neural Network (CNN) and Neural Architecture Search (NAS) [30,31,32,34,36,111]. Availability and ease of use for these tools vary. Currently, a web format and a command line tool are identified for all, except for Apindel, which mainly runs on a suite of python scripts. Some are currently not functional or run through a local application, which may pose a challenge for users preferring a direct web browser format.

These prediction tools focus on or reflect two major DNA repair pathways, C-NHEJ and/or MMEJ (Table 2). Prediction outcomes for Apindel, CROTON, SPROUT, Lindel, inDelphi, and FORECasT are applicable to template-free Cas9-mediated double-stranded DNA breaks [30,31,32,34,111]. More specifically, the most popular nuclease system utilized or trained on is SpCas9 which recognizes a ‘NGG’ PAM site. Since the discovery of SpCas9, smaller Cas have emerged and gained popularity as a substitute; however, prediction tools have not all broadened the range of Cas orthologues available or incorporated *Staphylococcus aureus* Cas9 (SaCas9) or CRISPR from *Prevotella* and *Francisella*1 (Cpf1(Cas12a)) which recognize ‘NNGRRT’ and ‘TTTV’ PAM, respectively [146,147]. However, inDelphi took a step in this direction and supports KKH SaCas9, a high-fidelity variant [34,147]. Cas nuclease type can significantly influence the DNA repair pathway and the resulting repair profile. Studies have suggested that SaCas9 is less prone to C-NHEJ +1 bp insertions than SpCas9. Yang et al. indicated that SaCas9 had ~10-fold fewer +1 bp insertion C-NHEJ editing alleles at the fourth nucleotide upstream of the PAM, in comparison to SpCas9 [148]. These results suggest these differences are due to the type of Cas utilized, further emphasizing the importance of providing Cas options in prediction tools.

Apindel, CROTON, SPROUT, Lindel, inDelphi, and FORECasT predict repair profiles such as insertions and deletions, and exclude substitutions, translocations, and inversions (Table 2) [30,31,32,34,111]. Predicting repair outcomes, such as the occurrence of a frameshift, is valuable in the goal of achieving gene knockouts or disruption of protein function resulting from a CRISPR/Cas9 application as discussed previously under repair outcomes. Frameshift frequency, fraction or ratio is estimated by Apindel, CROTON, SPROUT, Lindel, inDelphi, and FORECasT. It is important to note that although predicted editing outcomes are measured this way, they do not necessarily capture different functional outcomes. For example, studies addressing the success of therapeutic HIV-1 proviral targeting approaches are dependent on whether the repair outcomes result in proviral excision or inactivation [149]. Incomplete editing may result in partially intact proviral genomes or replication competent viral fragments that can persist [150]. These unresolved challenges highlight how editing outcomes may appear successful at the genomic level, but may not indicate success biologically or therapeutically. Editing results may be relevant beyond cell types, yet studies have indicated that some cell types may have major defects in DNA repair, impacting repair and creating variation in pathway efficiency [34,101,111,151]. A limitation of prediction frameworks is in the context of HIV-1 proviral targeting. The optimization of these models involve genomic systems such as reporter constructs and controlled editing conditions that may not fully capture the biological and structural complexity of DNA editing in a latent human reservoir. HIV-1 reservoirs are distributed across multiple cellular compartments, each with distinct DNA repair activities and chromatin landscapes [136,152,153]. As mentioned above, integrated HIV-1 provirus occupies heterogeneous locations on the human chromosome where chromatin accessibility can affect Cas ability to access the provirus [119,136]. These regions may differ in how transcriptionally active they are and are also affected by epigenetic regulation [136,152]. This can affect both cleavage efficiency and downstream DNA repair pathway under consideration and as a result predictions made from the less complex datasets may not accurately reflect editing outcomes observed at proviral human integration sites. Apindel, SPROUT, Lindel, inDelphi, and FORECasT include prediction of edits in human cell types, with SPROUT uniquely including primary human CD4+ T cells, a known HIV-1 reservoir in humans, and trained on endogenous loci compared to synthetic constructs and therefore may be more biologically relevant for modeling repair outcomes in the context of these reservoirs [30,32,34,110]. Additionally, FORECasT and inDelphi include a mouse cell line and FORECasT includes a hamster cell line [30,34]. Depending on the cell type planned for use in an experiment, or if the frequency of the different repair edits is a focus, users may want to take into account which prediction tool to utilize, as different tools may perform differently with specific cell types. Although challenging, taking into consideration and linking predictive modeling to biological outcomes may address editing strategies. Prediction tools play an important role in providing access to predicting CRISPR/Cas9 repair profiles and outcomes, contributing to improved understanding and a better design of gene editing experiments for a more precise, controlled approach [30,31,32,34,110,111].

### 4.2. Analytical Tools

The development of sophisticated computational analytical tools has been instrumental in advancing the study of CRISPR/Cas9 genome editing, providing researchers with powerful means to interpret complex experimental data. The ability to analyze and compare the outcome of experimental data is critical for understanding CRISPR/Cas9-induced repair profiles and outcomes. Although predictor tools are valuable, indel profiles can vary between target sites and are affected by delivery methods; therefore, reliable tools for downstream experimental analysis, which we are referring to as “analytical tools”, are needed to interpret results (Table 3 and Table 4) [42,154]. Understanding experimental outcomes addresses the concern of unintended mutations and disruption of essential genes, understanding favored DNA repair pathways, optimization and safety of CRISPR application, and more. The overall goal of various analytical tools and platforms that exist is to identify induced repair mechanisms or editing outcomes based on sequencing data generated from CRISPR/Cas experiments. Available tools vary in their implementation, and include a user-friendly web-based tool, a command-line tool/software package, or both (Table 3 and Table 4). Within tools considered in and at the time of this review, seven provide a web-based version, nine provide access to scripts via GitHub or Bioconductor, one upon request, with four providing both (Table 3 and Table 4) [29,35,36,37,40,142,143,144,145].

Configuration features may contribute towards the user’s choice of a desired tool. Therefore, developers should consider consistent maintenance and clear documentation to support usability. Although the timeframe of the latest web-based tool update is not always apparent, tools such as CRISPResso2, ampliCan, Rational InDel Meta-Analysis (RIMA) v1 updated into v2, PEM-Q, and CrispRVariants, indicated their most recent commit or script-based update was 2024 and later (Table 3 and Table 4) [29,36,39,142,144]. A gap in current analytical frameworks is the lack of standardized or unified benchmarking of datasets across tools, which differ in capabilities and are context dependent [139,141]. This variability makes it difficult to objectively compare accuracy and sensitivity across experimental conditions [139,143,145]. Although benchmarking is commonly performed using reference datasets, these are not consistently standardized across studies, and evaluation strategies may differ between synthetic control systems and endogenous loci [139,141,145,155]. This makes it difficult to determine which tools perform best in biological contexts such as primary cells, transformed cell lines, and in vivo systems [141,156,157].

**Figure 4:**
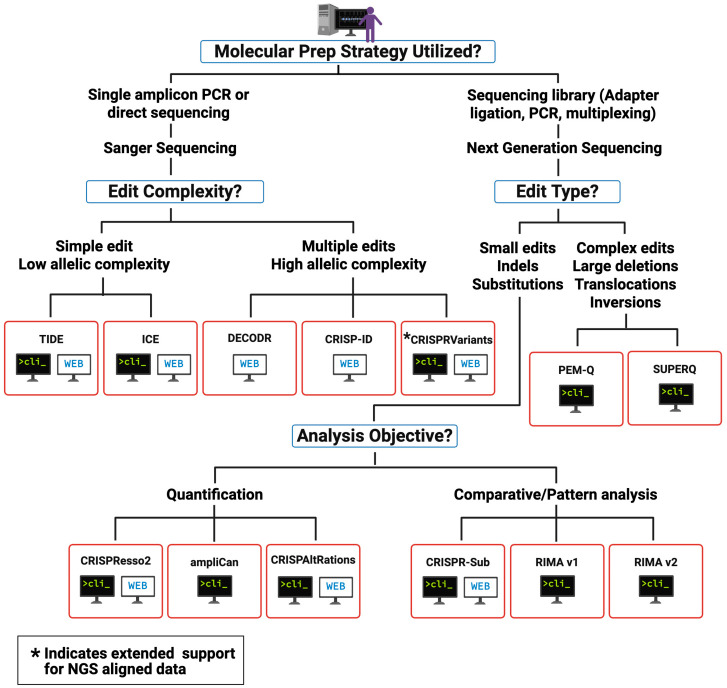
Decision framework for selection of analytical tools for CRISPR/Cas9 editing outcome analysis. The decision tree suggests and organizes commonly used analytical tools for the evaluation of CRISPR/Cas-induced repair profiles and outcomes. This takes into consideration the primary sequencing modality (Sanger sequencing vs. Next generation sequencing (NGS)). Sanger-based approaches primarily involve deconvolution of mixed chromatogram signals and tools are grouped by their ability to resolve simple edits with low allelic complexity, or multiple edits with high allelic complexity. NGS-based approaches primarily involve aligning sequencing reads and are divided into pipelines of editing outcomes geared towards small edits (indels/substitutions) or complex edits (large deletions/translocations/inversions). They are then further subdivided into primary quantification pipelines to measure editing efficiency/indel frequency, or statistical comparative or meta-analysis of editing profiles across samples or conditions. Tools are further differentiated into command line interface (CLI) or web–based platform capabilities indicated by developer resources. Together, this framework provides a suggested guide for selecting appropriate tool approaches for CRISPR/Cas9 editing evaluation. It is not intended to be restrictive. The included tools reflect approaches selected based on their described functionality and suitability for distinct analytical contexts defined by sequencing modality, edit type and complexity, and downstream analysis objectives. Note: * Indicates tools extended support for NGS aligned data. Created in BioRender. Dampier, W. (2026) https://BioRender.com/am70nme.

In the analysis of CRISPR/Cas DNA editing outcomes, no single analytical approach captures the full spectrum of possible edits. Interpreting these outcomes requires considering multiple dimensions, which can be broadly organized into categories. One category is the biological nature of the edit, which includes the edit type and its associated complexity or scale. Edit types span from small edits such as insertions, deletion, and substitutions to more complex edits or larger events such as large deletions, inversions, and translocations. A second category is the overall allelic complexity of the editing outcome. This spans from simple edits with low allelic diversity to more complex outcomes where the outcome is multiple edits and a range of edit sizes results in greater allelic heterogeneity (Figure 4) [35,36,51,139,141]. Commonly used amplicon-based tools are primarily optimized for detecting small local indels which are likely associated with the C-NHEJ repair pathway, which introduces a bias toward under detection of more complex repair outcomes [158,159,160]. Consequently, pipelines risk oversimplifying the biological consequences of CRISPR/Cas editing. In contrast, tools such as PEM-Q and SUPERQ (Table 3) are designed to resolve complex or long-range repair outcomes including structural rearrangements, providing a more comprehensive characterization of CRISPR-induced edits (Figure 4) [35,36]. Regarding edit complexity, samples containing multiple edits or alleles often produce overlapping signal peaks in Sanger sequencing outputs [161,162]. This increased signal complexity necessitates the use of deconvolution-based analytical tools such as TIDE, ICE, DECODR or CRISP-ID (Table 4), which attempt to resolve mixed editing populations (Figure 4) [40,140,143,145]. These approaches may lose accuracy when editing outcomes are highly diverse.

All analytical tools are capable of primarily interpreting downstream next-generation sequencing (NGS) (Table 3) or Sanger sequencing (Table 4) output from double-stranded DNA cuts, with the accepted input file type being a key feature that affects their usability and integration into analysis workflows (Figure 4). Analysis using Inference of CRISPR Edits (ICE), Deconvolution of Complex DNA Repair (DECODR), CrispRVariants, CRISP-ID, and Tracking of Indels by Decomposition (TIDE) all support input as AB1 file format (Table 4) [40,143,145]. This file type is produced from Sanger sequencing, also known as the gold standard for DNA sequencing due to its high accuracy and reliability through production of long reads up to 1000 bp [161,162,163]. Furthermore, Standard chromatogram format (SCF) files, also generated from Sanger sequencing, are supported as input by CRISP-ID and TIDE (Table 4) [140,145]. FASTQ files are a standard output of NGS also referred to as deep sequencing, which in contrast to Sanger sequencing, can perform sequencing of millions of small fragments of DNA in parallel [161,164,165]. AmpliCan, CRISPAltRations, CRISPR-Sub, CRISPResso2, SUPERQ and PEMQ read FASTQ files, with PEMQ created to specifically work with results from primer-extension-mediated sequencing (PEM-seq) (Table 3) [29,35,37,51,139,141]. During downstream NGS analysis, a variant table is created which RIMA v1 uses as an input file (Table 3) [29]. The more recent version, RIMA v2, can process CRISPResso output to generate these variant tables in a format compatible with this Microsoft Excel for Microsoft 365-based tool (Table 3) [29,144]. Tools available support analysis of experiments as individual and/or batch which is useful for analyzing multiple files and/or experiments simultaneously. Tools that identify both individual and batch file input include ICE, DECODR, CRISPR-Sub, ampliCan, CRISPAltRations, CRISPResso2, which contains CRISPRessoBatch as part of their suite of complementary tools, RIMA v1, RIMA v2, and TIDE (Table 3 and Table 4) [29,37,39,40,143,144,145].

The capabilities of CRISPR/Cas9 gene editing include the use of multiple gRNAs, overall expanding the scope of CRISPR/Cas9 applications to improve editing efficiency, specificity, and more. Therefore, user interest exists in the ability of a tool to accurately interpret more complex editing outcomes resulting from multiple gRNA experiments. Tools that indicated supporting the analysis of multiple gRNAs include ICE, DECODR with a maximum of two, CRISPResso2 for batch processing, TIDE, ampliCan, and CRISPAltRations [39,40,51,139,143,145]. In the context of multiplex editing, performance differences between tools become more pronounced. Amplicon-based tools such as CRISPResso2 and ampliCan perform well for quantifying editing efficiency and indel frequency at defined loci but are limited in reconstructing large deletions across multiple cut sites (Figure 4) [35,36,139,141]. Tools such as PEM-Q and SUPERQ are analysis frameworks developed for the analysis of PEM-seq-derived datasets and sequencing approaches focused on repair junctions that capture dsDNA break repair outcomes [35,36]. These tools are better suited for identifying excision events and rearrangements, making them more appropriate for applications such as proviral HIV-1 excision strategies where studies use two or more gRNAs [27,35,36,166,167].

Manual interpretation of repair profiles and specific outcomes is possible; however, some analytical tools identify these features, reducing the need for user manipulation of sequencing output. Tools examined in this review identified insertions and deletions (Table 3 and Table 4) [29,35,39,142,143,144,145]. Cas-mediated substitutions are explicitly quantified or reported by tools such as CRISPR-Sub and CRISPResso2 (Figure 4) (Table 3) [29,37,39,51,139,141,144]. In CRISPR/Cas9 experiments, substitutions are sometimes filtered out to minimize sequencing noise [168]. This introduces the challenge of true biological substitutions, particularly in highly variable systems such as HIV-1 proviral genomes, may be misclassified as sequencing errors [155]. Distinguishing true CRISPR/Cas-induced substitutions from background variation is essential since HIV-1 quasi-species diversity can complicate accurate identification of editing outcomes [37,141,169]. Tools with sufficient sensitivity to retain and quantify substitutions provide an advantage in more accurately capturing variant profiles and genome editing outcomes [139]. SUPERQ and PEMQ both identify translocations, and PEMQ also identifies inversions (Figure 4) (Table 3) [35,36]. These differences highlight that tools are optimized for local indel detection, while a subset captures larger rearrangements, limiting comprehensive detection of complex repair events (Figure 4). As mentioned for predictive tools, identifying repair outcomes such as a frameshift mutation, or their likelihood, is a valuable feature in analytical tools. DECODR, ampliCan, CRISPResso2, CRISPAltRations, CrispRVariants, TIDE, and ICE (based on a knockout score) are among these tools (Table 3 and Table 4) [39,40,51,139,142,143,145].

CRISPResso2, CRISPAltRations, and ampliCan perform well for quantifying on-target edits within predefined regions (Figure 4) and are primarily designed for this purpose rather than genome wide off-target discovery [51,139,141]. This constraint is important in therapeutic applications where comprehensive identification of unwanted edits is critical for safety [170,171]. As a result, analysis must be complemented by genome wide or targeted sequencing approaches. This can include in silico predictions followed by experimental methods like GUIDE-seq [171,172,173]. This reliance on separate workflows highlights a gap in the integration between whole genome off-target detection and downstream quantification. Beyond tool-specific considerations, a limitation is the reliance on a single reference sequence. This can mask sequence heterogeneity within experimental samples. This is especially important in applications such as HIV-1 proviral targeting where genetic diversity within a single host can exist [169,174]. Pipelines that do not account for this variability may underestimate editing diversity or misinterpret repair outcomes.

Despite widespread application of CRISPR/Cas9, many studies do not explicitly report the use of a downstream analytical tool, pushing tool performance to be highly context dependent. Sanger-based approaches such as ICE, are effective and well suited for rapid and low-throughput validation, complementing NGS-based methods which provide higher sensitivity [141,143]. Amplicon-based tools (Ex. CRISPResso2, ampliCan, CRISPAltRations) provide robust quantification of local indel frequencies and editing efficiency but are less suited for detecting large deletions or complex rearrangements (Figure 4) [51,139,141]. In contrast, specialized pipelines (Ex. PEM-Q, SUPERQ) outperform standard approaches in resolving repair junctions and structural variants, although they require more complex experimental design and are less accessible for routine analysis [35,36]. Tools such as RIMA and CRISPR-Sub offer advantages for comparative statistical analyses, building on upstream pipelines, although they do not resolve limitations on primary edit type detection [29,37,144].

Collectively, current analytical frameworks reflect a trade-off between scalability and sensitivity. High-throughput amplicon-based tools are better suited for quantification of simple edits, whereas specialized approaches more effectively detect complex edits [35,36,51,139,141]. A key challenge is the lack of integrated frameworks that can capture small variants with high sensitivity and reliably detect large scale or multiple edits across diverse biological systems. Addressing this gap may improve benchmarking and reduce analytical bias. While certain analytical tools discussed may not differentiate among specific end-joining (EJ) pathways, they are powerful and helpful for detecting CRISPR/Cas9 editing events through the identification, quantification, and visualization of mutations. The selection of the most appropriate tool should be guided by the analytical requirements of the study and needs of the user.

## 5. CRISPR/Cas9 Strategies for an HIV-1 Cure

HIV-1 currently has no FDA-approved cure. The infection can be effectively managed with antiretrovirals (ARTs) [149,175,176,177]. ARTs target various stages of the HIV-1 replication cycle. However, none of the currently approved ARTs target the integrated HIV-1 provirus [149,173,177]. The HIV-1 provirus persists throughout the lifespan of the infected cell, and this leads to the formation of reservoirs across the body of people living with HIV (PLWH) [149,177]. Studies have been ongoing for over a decade to develop therapies that target the integrated HIV-1 provirus. Some of the early attempts at targeting the integrated provirus involved the use of DNA editing tools like Tre-recombinases, TALENS, and zinc finger nucleases [175,178,179,180]. These DNA editing tools have been used to target the HIV-1 long terminal repeat (LTR), also termed the promoter in HeLa cells (Tre-recombinases) and the TAR region of the provirus in the Jurkat T cell line (zinc finger nucleases) [178,179]. However, another gene editing tool, CRISPR/Cas9, gained traction because of the advantage of maintaining the same Cas protein with an easily programmable gRNA for different gene targets implicated in HIV-1 infection [175,180]. This made the CRISPR/Cas9 systems more scalable and allowed for multiplex targeting within the same HIV-1-infected cell [175,180]. In this section, we discuss different CRISPR/Cas9 strategies that have been used towards an HIV-1 cure.

### 5.1. HIV-1 Gene Targets and CRISPR/Cas9 Strategies

The ability of HIV-1 to mutate is a key attribute of the virus that spearheads its immune evasion and drug resistance mechanisms [177]. The genetic variability in HIV-1 genes is higher in some regions than others [181,182,183]. Reverse transcriptase, one of the encoded viral enzymes, is known to be error-prone and is mainly responsible for the introduction of mutations in the virus’s genome during viral replication [174,177,184,185]. In context, the *envelope (env)* gene is the most variable region, with the V1-V5 loops of HIV-1 gp120 contributing to its high genetic diversity [182,186,187,188,189,190]. The variability in the *envelope* gene facilitates immune escape and influences co-receptor usage [182,186,187,190,191]. Within the *env* gene, the gp41 ectodomain shows the least diversity compared to gp120 and the CTT domain of gp41 [192,193]. The variability in gp41 also contributes to immune evasion and viral tropism [193].

A more conserved viral gene region is *pol*, which includes *reverse transcriptase* (RT), *integrase* (IN), and *protease* (PR) [184]. Group M HIV-1 shows high amino acid conservation in IN (96.02%), RT (94.07%), and PR (93.11%) [184]. However, variations do occur within the *Pol* region, and they are associated with ART resistance mechanisms [184]. Protease has the most variation within *pol* [184]. Mutations in PR are associated with drug resistance mechanisms against protease inhibitors [194]. There are also drug resistance mutations against reverse transcriptase inhibitors, an indication of the selection of mutations in the *reverse transcriptase* gene [184,194]. Drug resistance mutations in PR and RT have also been identified in viruses isolated from treatment-naive individuals, making these mutant viruses an important public health concern [194]. *Integrase* has the least variation amongst the encoded viral enzyme genes [184,188]. In addition, there is a high genetic barrier to IN mutations against Integrase (IN) strand transfer inhibitors (INSTIs) (especially second-generation INSTIs) in some HIV-1 subtypes [195], with some IN mutations leading to a decrease (Q148H) or increase (G140S) in viral fitness cost [196].

The *gag* gene segment includes capsid, matrix, and nucleocapsid protein [194]. The most variable region is the *matrix* gene, where subtype-specific polymorphism exists [192,193]. The nucleocapsid and capsid are more conserved [197,198]. The LTR region of the HIV-1 provirus serves as a promoter for its structural, enzymatic, and accessory genes. The LTR region includes the R, U5, and U3 regions [191,199,200,201]. The U3 region is a commonly targeted region for HIV-1 CRISPR/Cas9 studies and has variable and conserved regions [199,202]. There are subtype and quasi-species differences at the NF-kB and Sp1 sites of the U3 region, while the TATA and TAR sites of the U3 and R regions, respectively, are more conserved [181,191,199,202,203,204].

**Figure 5:**
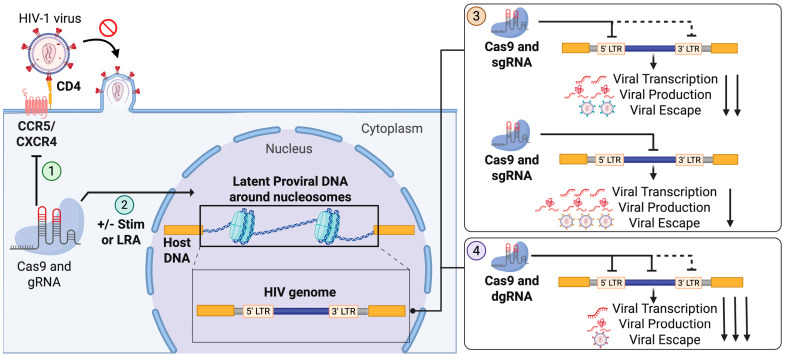
Conventional CRISPR/Cas9 strategies targeting the latent HIV-1 provirus. In vitro and in vivo CRISPR/Cas9 strategies against HIV-1 replication targeting human and or HIV-1 proviral genes. **1. CRISPR/Cas9 Human Targets.** CRISPR/Cas9 strategies targeting human genes involve HIV-1 coreceptors *CCR5* or *CXCR4*, or sometimes both. Knockout of these human genes leads to inhibition of HIV-1 entry **2. Stimulation of latently infected cells to reactivate HIV-1.** CRISPR/Cas9 strategies targeting the HIV-1 provirus in latently infected cells may require treatment with LRAs or stimulation (e.g., with TNF-α) to reactivate the HIV-1 provirus and enhance Cas9 activity. CRISPR/Cas9 strategies targeting different regions of the HIV-1 provirus may involve the use of gRNA in single or combinations. **3. CRISPR/Cas9 strategies involving sgRNAs.** Common CRISPR/Cas9 HIV-1 proviral targets include the LTR regions and non-LTR regions like *Gag* and *Pol*, and accessory genes like *Tat* and *Rev*. Single gRNAs targeting the LTR regions show an enhanced reduction in viral transcription, production, and escape compared to targeting non-LTR regions of the provirus. **4. CRISPR/Cas9 strategies involving dgRNAs.** A common combination gRNA strategy involves targeting the HIV-1 LTRs (5′LTR and 3′LTR) and other non-LTR HIV-1 proviral gene regions. This results in a greater reduction in viral transcription, production, and escape compared to single-gRNA strategies against the HIV-1 provirus. Created in BioRender. Urturi Ortiz, N. (2026) https://BioRender.com/bnhpbjs.

A study by Ebina et al. targeted the NF-kB and TAR sites of the U3 region and R region of HIV-1 LTR, respectively, in the HIV-1 latently infected Jurkat T cell line [205]. This led to the inhibition of GFP expression from the integrated HIV-1 construct in Jurkat cells [205]. This suggested the potential role CRISPR/Cas9 can play in achieving a functional HIV-1 cure. Since the Ebina et al. study, the HIV-1 LTR and other gene segments of the provirus have been targeted in several studies (Figure 5) [24,185,206,207,208]. Conserved regions within the HIV-1 LTR, like the TAR site in the R region, are attractive target gene regions for inhibiting HIV-1 transcription and replication, more so compared to other regions of the provirus like *Env*, *Pol*, and *Gag*, because targeting any region in the 5′ LTR will likely lead to targeting in the 3′ LTR region (Figure 5) [10,158,170,173,207,209,210,211]. Excision events spanning the cut site in both LTRs (5′ and 3′) and indels like deletions and insertions at both cut sites (5′ and 3′) have been identified after the U3 and R region targeting [27]. HIV-1 LTR targeting has been investigated in in vitro in cell lines like SupT1 [206,212], Jurkats [205], J-lats 10.6 [194], CHME5 [184,194], and TZM-bl [194]. These studies have also been conducted in primary and primary-like cells, like CD4 T cells from PLWH and IPSC-derived monocytes and macrophages, respectively [184,188].

Most of the repair edits after sgRNA CRISPR/Cas9 editing of the HIV-1 provirus in HIV-1-infected cells (i.e., CD4 T cells, monocyte-macrophages, and microglia) are indels of various sizes and nucleotide substitutions [195]. Amongst these repair edits, small-base pair deletions and insertions predominate, and these are likely associated with the C-NHEJ repair pathway [196]. Less frequent repair edits, which include large base-pair deletions, also occur and are likely associated with the MMEJ repair pathway [29]. Indels generated after CRISPR/Cas9 editing in HIV-1-infected cells are also dependent on factors like target sequence, time of editing, chromatin, and DNA repair pathways [29,101,103]. HIV-1 targeted cells are known to express proteins in the C-NHEJ, MMEJ, and HR repair pathways, and expression of these proteins can be impacted by activation [213,214]. Therefore, differential expressions of proteins in these DNA repair pathways can also impact the contribution of these DNA repair pathways after CRISPR/Cas9 editing, the repair profiles generated, and CRISPR editing efficiency. Stable delivery of CRISPR components (e.g., through lentiviruses) also enhances mature repair profile generation through longer Cas9 editing time and re-editing of repaired profiles, which promote larger deletions and likely reduce the chances of viral escape mutants [41,212]. Cas9 has also been shown to induce heterochromatin markers like H3K9me3 histone modifications at the cut site, thereby suppressing HIV-1 transcription [159].

Highly variable viral genomic regions can naturally select for CRISPR/Cas9 resistance or can accommodate repair profiles after CRISPR/Cas9 editing that reduce CRISPR/Cas9 efficiency and increase the selection of viral escape mutants with time (Figure 5) [206,212]. Interestingly, sequencing of viral escape mutants from HIV-1-infected SupT1 showed the presence of small base-pair indels and substitutions that cluster around the Cas9 cleavage site, an indication of C-NHEJ involvement in the generation of these escape viral mutants [44,207]. In contrast, there are fewer viral escape mutants with large base-pair deletions or insertions, an indication of less tolerance for these types of repair edits in these viral escape mutants [212]. The MMEJ pathway plays a role in the generation of large base-pair deletions, and therefore, it is likely that repair profiles with these MH-mediated deletions can reduce the chances of viral escape mutants. Therefore, regardless of the variation present in an HIV-1 proviral gene segment, identifying relatively conserved target sequences in that viral gene segment may help improve the status of that gene segment as a viable target region [207,211]. It is also important to note that this conservation needs to be there for both the PAM as well as the protospacer [9,10].

Another approach that incorporates targeting other non-LTR regions is the use of a dual or multiple gRNA approach (Figure 5) [160,206]. Dual or multiple gRNA CRISPR approaches have been shown to improve inhibition of proviral transcription and/or reduce reporter expression while impeding the selection of HIV-1 escape mutants (Figure 5) [160,215,216]. Multiplexing of gRNAs also enhances excision events between two or multiple cut sites [160,206]. Though dgRNA use has been shown to enhance CRISPR/Cas9 editing and reduce or prevent breakthrough viruses, the individual gRNA used in this multiplex approach matters. Less optimal gRNA combinations are not efficient in preventing viral escape compared to more optimal gRNA combinations [206]. Optimal gRNAs are individually able to delay viral escape mutants and have a synergistic effect when used in combination [206].

The success of the CRISPR/Cas9 tool against the HIV-1 provirus in vitro necessitated the progression of these studies into animal models [27,28,170,208]. Some of these studies have been conducted in HIV-1-infected humanized mice [27] and SIV-infected non-human primates [28]. Observations from these in vivo CRISPR/Cas9 studies, especially with the use of two gRNAs, showed HIV-1 provirus excision events in multiple tissues, which serve as viral reservoirs [27,28]. These HIV-1/SIV-infected animals also showed improved CD4 T cell counts and a reduction in their viral load [27,28,215,216]. Unfortunately, viral rebound occurred in many of the HIV-1-infected and CRISPR-treated animals at later timepoints post ART withdrawal [27]. Some of the possible explanations for this rebound include a less efficient CRISPR component delivery into all HIV-1 reservoirs and the possible selection of viral escape mutants [24,207]. However, some animals had not rebounded after the 6-month time point, providing a possibility for this approach [27]. Given this, evidence in vivo and in vitro of HIV-1 transcription and replication inhibition has spearheaded this anti-HIV-1 CRISPR-based approach into Phase I clinical trials (NCT05144386).

### 5.2. Human Gene Targets and CRISPR/Cas9 Strategies

HIV-1’s gp120 utilizes CCR5 (R5-tropic virus) or CXCR4 (X4-tropic virus); both of these co-receptors are used for viral entry after initial binding to CD4 on the surface of permissive cells [175,217,218]. Both co-receptors share a number of similar features [217]. They both have N-terminal extracellular domains, seven transmembrane alpha-helixes, three extracellular loops, three intracellular loops, and C-terminal intracellular domains [219,220,221]. The extracellular loop 2 (ECL2) is involved in ligand and HIV-1 gp120 binding, while the intracellular loops are involved in G-protein coupling and intracellular signaling [217,219,221]. Though CCR5 and CXCR4 are structurally similar, they have a protein sequence identity of about 34% and possess differences in regions like the extracellular and intracellular second loops (ECL2 and ICL2) [217,222]. A small number of individuals within the human population possess a 32-base-pair deletion in exon 3 (which affects ECL2) of the *CCR5* gene [175,221,223,224]. This mutation leads to a frameshift mutation, which hinders the expression of CCR5 on the surface of the HIV-1-infected cells [220,225]. Reduced expression of CCR5 then blocks the required step of co-receptor binding by the variable region of gp120, inhibiting viral entry. This makes these select individuals resistant to R5-tropic HIV-1 infection [220,223].

Though there is no approved cure for HIV-1, there have been recognized cases of cured PLWH [226,227,228,229,230]. These include the cases of the Berlin, Düsseldorf, and London patients, where hematopoietic stem cells possessing the CCR5 Δ32/Δ32 mutation were transplanted into these individuals after whole body irradiation to treat their underlying cancer and HIV-1 diagnosis [5,221,226,231]. The relevance of these HIV-1 co-receptors in viral entry and these cases of an HIV-1 cure have been the basis of CRISPR strategies targeting CCR5 and CXCR4 in cells permissive to HIV-1 (Figure 5) [5].

CRISPR/Cas9-mediated CCR5-knockout strategies have included the targeting of specific sequences in regions like exon 1 and 3, leading to the disruption or the introduction of the CCR5 Δ32/Δ32 mutation in CCR5 [232,233,234]. Another important strategy in targeting the *CCR5* gene is to ensure the target sequence has little to no homology with the *CCR2* gene [223,235]. CCR5 shares 66% protein sequence identity with the C-C chemokine receptor, CCR2 [236], and therefore several studies have ensured that CCR5 targets have no homology with CCR2. This is done in conjunction with screening for off-targets genome-wide [223,235,236]. The success of CRISPR/Cas9-mediated CCR5-knockout strategies has been demonstrated in both mammalian cell lines [234,235], primary-like cells [223], primary cells [237], and animal models [233,238]. CRISPR/Cas9-mediated CCR5-knockout strategies have been successful in enhancing resistance to R5-tropic HIV-1; however, there are reports of drawbacks to this strategy [220]. Some studies show that the CCR5 Δ32/Δ32 mutation in CCR5 increases susceptibility to viral infections like influenza and more fatal ones like West Nile virus infection [220]. Therefore, more studies are required to assess this important limitation in targeting CCR5.

CXCR4, the other co-receptor used by HIV-1 for viral entry, has also been targeted as a strategy against HIV-1 infection, although in fewer studies compared to CCR5 [175,232,239]. Gene disruption has been achieved in *CXCR4* through targeting regions like Exon 2 of the CXCR4 [232,239]. Gene disruption in *CXCR4* also led to the inhibition of X4-tropic HIV-1 in HIV-1-infected cells [232,239]. CXCR4 plays a role in keeping hematopoietic stem and progenitor cells (HSPCs) in the bone marrow [239,240]. CXCR4 pharmacological antagonism has also been shown to mobilize HSPCs in the bloodstream out of the bone marrow [240]. This potential limitation of CRISPR-mediated *CXCR4* targeting, therefore, requires further investigation in enhancing *CXCR4* as a viable target [239,240]. There have also been CRISPR/Cas9 strategies that involved targeting both *CXCR4* and *CCR5* [224,225,232]. This provided resistance to both X4 and R5 tropic HIV-1 [224,225,232].

**Figure 6:**
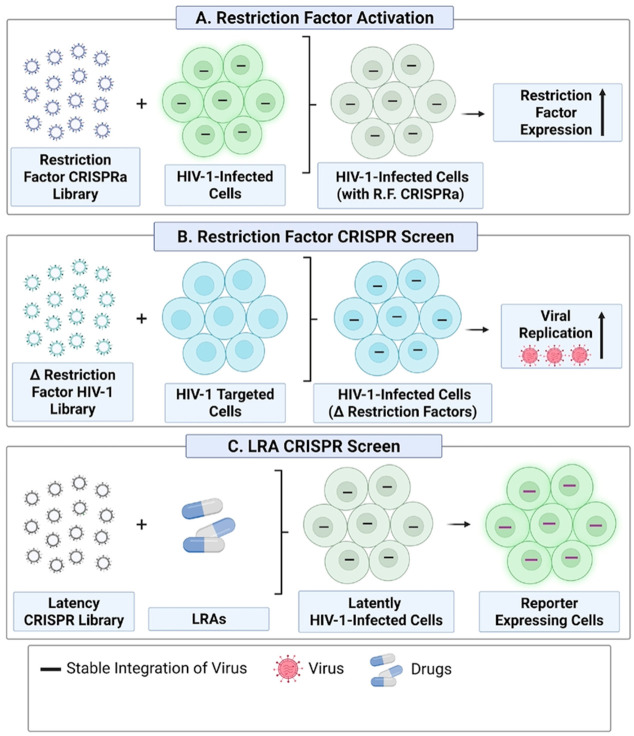
CRISPR/Cas9 screens and strategies towards inhibition of HIV-1 replication. This schematic illustrates CRISPR/Cas9-based screens or strategies designed to identify host genes involved in the regulation of HIV-1 latency and inhibition of HIV-1 replication. (**A**) **Restriction factor activation:** A CRISPR activation (CRISPRa) library that induces expression of restriction factors (R.F.) like APOBEC3B, which are lowly expressed in HIV-infected cells to inhibit HIV-1 replication. (**B**) **Restriction factor CRISPR screen.** A CRISPR/Cas9 restriction factor knockout (Δ) replication-competent HIV-1 library was used to screen restriction factors involved in the inhibition of HIV-1 replication in HIV-1 targeted cells. Knockout of these restriction factors leads to increased viral replication. (**C**) **LRA CRISPR screen.** A CRISPR/Cas9 knockout library targeting host genes associated with HIV-1 latency is combined with suboptimal doses of LRAs in latently HIV-infected cells. The synergistic effects of knockout of these host genes and suboptimal doses of these LRAs lead to enhanced reactivation of the HIV-1 provirus or increased expression of the HIV-1 construct (e.g., increased expression of a reporter gene like *GFP*) in HIV-1-infected cells. Created in BioRender. Dampier, W. (2026) https://BioRender.com/ah0zi15.

Cellular restriction factors (or antiviral factors) are another example of human gene factors that have been investigated as possible druggable or targetable candidates after CRISPR screens reveal their impact on HIV-1 replication (Figure 6) [241,242,243]. Some of the candidates identified to be involved in the inhibition of HIV-1 replication include *GRN*, *CIITA*, *APOBEG3G*, *SERINC5*, and *IFI16* [241,242,243]. Further investigations will be required to determine whether these cellular genes are viable targets either through pharmacological inhibition of accessory proteins targeting these restriction factors [242], or CRISPR gene knockout of HIV-1 accessory proteins targeting these restriction factors [241,242], or overexpression of these restriction factor genes like *APOBEC3B* (Figure 6) [243].

### 5.3. Other CRISPR/Cas9 Strategies Against HIV-1 Infection

Aside from the above-mentioned human and viral targets, there are CRISPR-based strategies targeting HIV-1 latency [149,166,167]. Certain CRISPR targetable regions of the HIV-1 provirus, like the 5′-LTR, can be embedded in a closed chromatin environment [136]. This can reduce Cas9 binding and HIV-1 proviral gene editing efficiency [103,119,132]. Shock and kill strategies have been used to reverse HIV-1 latency to reactivate proviral transcription and enhance immune recognition of infected cells [136,149,183]. Latency reversal agents (LRAs) like histone deacetylase inhibitors (e.g., Trichostatin A) have long been used to achieve HIV-1 latency reversal [136,166,167]. However, there are several limitations associated with these agents. LRAs that can target every HIV-1 reservoir across the body of PLWH remain elusive [244]. Adverse drug reactions associated with these drugs further complicate their use, as some of these LRAs are said to have different mechanisms of action [244]. To address this problem, a study by Dai et al. used CRISPR screens to determine which gene knockouts lead to synergistic latency reversal with LRAs (Figure 6) [244]. This screen, which also involved primary cells from PLWH, helped identify genes like *YPEL5*, which are involved in HIV-1 latency. This creates opportunities for the development of novel LRAs to target products of these genes. Again, different combinations of LRAs targeting different HIV-1 latency gene products and administered at suboptimal doses could be a potential strategy for enhancing HIV-1 reactivation or latency reversal (Figure 6) [244]. This strategy will require immune recognition and killing of productively infected cells [244].

**Figure 7:**
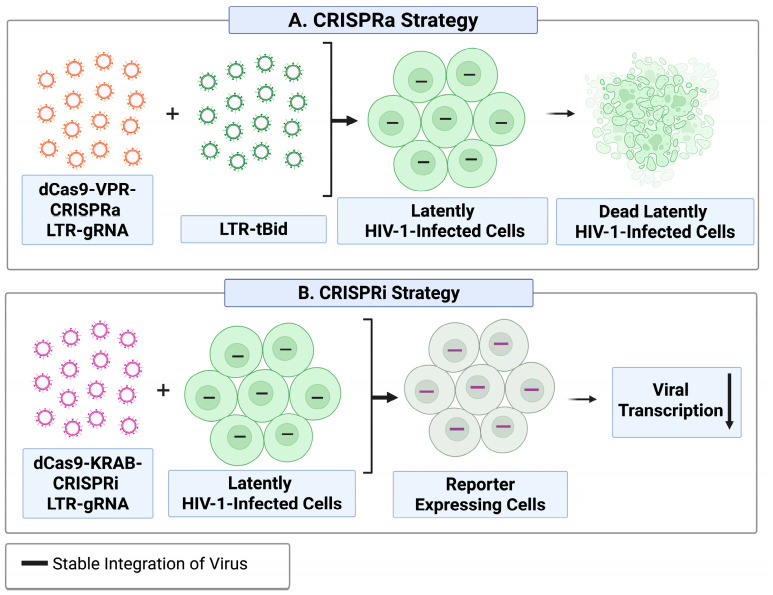
CRISPRa and CRISPRi strategies towards inhibition of HIV-1 replication. This schematic illustrates CRISPR/Cas-9-based strategies designed to induce programmed cell death of HIV-1-infected cells and suppress proviral gene transcription. (**A**) **CRISPRa strategy:** A CRISPR activation strategy which employs the use of dCas9 fused with an activation domain and combined with an LTR gRNA and an LTR-controlled suicide gene, like *tBid*, to suppress productive HIV-1 replication by enhancing cell death among the infected cells. (**B**) **CRISPRi strategy:** A CRISPR interference strategy which employs the use of dCas9 fused with a transcriptional repressor domain, like KRAB, and combined with a gRNA targeting the HIV-1 LTR, to suppress active proviral gene transcription. Created in BioRender. Dampier, W. (2026) https://BioRender.com/120vdrx.

Other LRA/CRISPR/Cas9 strategies are less dependent on immune killing of infected cells [166,167]. These strategies involve LRAs and CRISPR/Cas9 targeting of the provirus [166,167]. Another HIV-1 latency reversal strategy that does not involve immune recognition of productively infected cells involves the use of an inactive Cas9 (dCas9) fusion protein with transcriptional activation domains which does not generate DNA DSBs [245]. In a simultaneous viral transduction, a CD3-targeting adenovirus expressing a dCas9 fusion protein and an LTR-targeting gRNA (CRISPRa) and an adenovirus expressing an LTR-controlled truncated Bid (*tBid*) suicide gene were used to infect J-Lat 10.6 cells (Figure 7A) [245]. This led to reduced productive HIV-1 replication and enhanced cell death among infected cells [245]. These strategies highlight the possibilities of targeting both viral and human targets in the search for a comprehensive cure for HIV-1. In another study involving dCas9, this time fused with a transcriptional repressor domain (KRAB), and used in conjunction with a gRNA targeting the R region of the HIV-1 LTR, it was shown to repress active proviral gene transcription compared to when the gRNAs targeted other regions of the provirus (Figure 7B) [158,246]. Lastly, since DNA DSBs are not associated with the use of dCas9, the potential for off-target effects is reduced thereby enhancing the safety of this technology [247]. Taken together, these strategies expand the potential use of CRISPR/Cas9 technology outside the canonical use of this technology (direct targeting HIV-1 viral gene and/or host genes like *CCR5* and *CXCR4*) in targeting the latent HIV-1 reservoirs.

## 6. Concluding Remarks

The advances and extensive utilization of CRISPR/Cas-based systems make it the most prominent gene editing tool available for gene therapy. Over the past decade, CRISPR/Cas9-based gene editing tools have been used to achieve efficient gene knockout in vitro and in vivo [25]. gRNA multiplexing has facilitated multiple gene targeting and enhanced CRISPR/Cas9-mediated gene knockouts [25]. CRISPR/Cas9 screens have also identified druggable new targets implicated in disease conditions [6,20,241,244]. These advances have led to the first approved CRISPR-based therapy for the treatment of sickle cell anemia and transfusion-dependent beta thalassemia [19,23]. Despite these successes, the next decade will be critical as we continue to advance this technology to treat and or cure chronic genetic, metabolic, and infectious diseases. What is done with this technology also matters, as several ethical concerns and objections stand to derail progress made to deliver the much-needed treatment to patients [248,249,250]. The next decade should optimistically see an increase in approvals for CRISPR-based therapies, which have better delivery systems and have minimal off-target effects [25]. However, from a regulatory perspective, regardless of the strategies that may be developed to enhance CRISPR/Cas9 editing efficiency, there must be confidence in the reproducibility of these efficiencies and off-target effects must be consistently proven to be minimal. Reproducibility in efficiency and safety must also be strongly tied to a strong GMP framework with a clear indication of the clinical use of these therapies.

This review focuses on some of the factors that will impact the success of these CRISPR-based therapies going forward. Cas9 binding and efficiency, repair profiles, and repair outcomes determine the success of CRISPR/Cas9-mediated gene knockouts or manipulations. Cas9 binding and efficiency are influenced by factors like the chromatin environment surrounding the target sequence, presence or absence of mismatches in seed sequence (10–12 base pairs proximal to PAM site), Cas9 and gRNA abundance, and the specificity of the gRNA(s) [21]. Cas9 efficiency has also been shown to improve with high-fidelity Cas variants like HiFi Cas9 and Cas9_R63A/Q768A [25]. Repair edits like Indels are influenced primarily by the target sequence, and other factors like time, cell cycle, chromatin environment, and DNA DSB repair pathways and proteins [4,65,101,103,125]. However, the repair outcomes (i.e., whether in or out of frame mutations) can be determined by the length of the indel profiles generated. Insertions and deletions of length 3 or multiples of three base pairs are likely to be in-frame mutations and will likely not alter the coding region of the target gene, while indel lengths outside this parameter will likely lead to frameshift mutations or out-of-frame mutations [29,30].

Machine learning tools are another important piece that will drive the success of CRISPR-based therapies. These tools are based on algorithms that help analyze and/or predict repair profiles and outcomes, and off-target effects to boost the efficiency and ensure the safety of these therapies [29,30,31,41]. Taken together, in our quest to enhance CRISPR/Cas9 editing efficiencies, predictive computational tools can provide firsthand in silico evidence of efficacy and off-target effects. This should help rationalize and develop repair profile and outcome modulatory strategies based on the factors discussed in this review. In the context of HIV-1, some of these strategies could include (1) in silico prediction and target evaluation experiments to ensure sequence targets are conserved across HIV-1 clades and that they are not prone to in-frame mutations; (2) modulation of the DNA repair pathway, such as the C-NHEJ, to enhance deletions that may be inactivating to the provirus while assessing the impact on off-target effects; and (3) modulation of the chromatin environment to increase openness and enhance CRISPR-Cas9 editing of the HIV-1 provirus.

These factors come into play after the successful delivery of CRISPR/Cas9 into target cells. As we move more of these CRISPR/Cas-based therapies from bench to bedside, we are met with challenges outside CRISPR/Cas9 activity. The journey of CRISPR/Cas components is fraught with many obstacles after delivery in vivo. In blood, these components can be degraded, opsonized, or phagocytosed by immune cells, or there can be a failure to extravasate the blood vessel [24,25]. If they extravasate the blood vessel intact, they must deal with the possibility of extracellular matrix entrapment or local tissue confinement [24,25]. When they do reach the target cell, there can be failure to release the CRISPR/Cas components into cells, or they can be degraded within the host cell [25]. Therefore, cell and tissue architecture are important considerations in the development of optimal delivery systems for CRISPR/Cas9 components [24]. Taken together, CRISPR/Cas-based gene editing technologies have transformed the field of gene therapy, and therefore, further advancement in these therapies will likely hold the key to the treatment or cure of a lot of diseases that otherwise could have been neglected, debilitating, or fatal.

## Figures and Tables

**Table 1:** Repair profiles, repair outcomes, and computational tools. This table provides a list of studies that have investigated repair profiles after CRISPR/Cas9 editing. The table is organized to provide information on the authors of the study, the study structure which includes the delivery of CRIPSR components, and the cell models used, the repair profiles after CRISPR/Cas9 editing, the repair profile sizes (with particular focus on indel sizes), and the outcomes of these repair profiles, as well as the prediction or analytical tools used in the study.

Study	Study Structure	Repair Profile	Repair Profile Size	Repair Outcome	Computational Tool
Taheri-Ghahfarokhi, A. et al. [29]	In vitro Transient expression of SpCas9 and gRNA in HEK293 (Plasmid-FuGENE transfection). DNA extraction-72 h after transfection	Deletions (55.57%)	1 bp (14.85%) 6–10 bp (22.28%) 11–20 bp (38.07%)	In-frame (16.28%) Frameshift (83.72%)	RIMA
		Insertions (41.02%)	1 bp (93.47%) 2 bp (6.53%)		
Allen, F. et al. [30]	In vitro gRNA Lentivirus transduction of Cas9-expressing K562 cells. DNA extraction-7 days after transduction/selection	Deletions (76%)	1 or 2 bp (18%) >2 bp (58%)	Observed in-frame and out-of-frame mutations	FORECasT
		Insertions (16%)	1 bp (13%) >1 bp (3%)		
Leenay, R.T. et al. [31]	In vitro SpCas9 RNP electroporation into primary T cells. DNA extraction-6 days after electroporation	Deletions (31%)	Av. length (13 bp)	Prediction of likely and least likely frameshift outcomes	SPROUT
		Insertions (20%)	1 bp (95%)		
Chen, W. et al. [32]	In vitro gRNA lentivirus transduction of SpCas9-expressing HEK293T cells. DNA extraction—5 days after transduction	Deletions (63.6%)	Majority were small bp 25 to 150 bp (1.5%)	Observed and measured frameshift ratios were high and correlated	Lindel
		Insertions (31.5%)	Majority were 1 bp		
Chakrabarti, A.M. et al. [33]	In vitro gRNA Lentivirus transduction of Cas9-expressing HepG2 cells. DNA extraction-5 days after transduction	Insertions and Deletions observed	+ 1 bp insertion (44%); +1 bp deletion (26%) Larger deletions were also observed	81% of indels cause frameshift mutations. Certain sites show a strong preference for in-frame indels	Artificial neural network
Shen, M.W. et al. [34]	In vitro mESC with stable integration of guideRNA and target sequence (Lib-A), then transfected with Tol2 transposon-based SpCas9 expression plasmid. DNA extraction after 7 days	Deletions (87%) Insertions (13%)	MH deletions (58%) MH-less deletions (25%) 1 bp insertion (9%) Others (8%)	MH deletions in human exons tend to be in-frame. Cas9-mediated deletions were used to correct pathogenic frameshift mutations in disease alleles	inDelphi
Yin, J. et al. [35]	In vitro HEK 293 Transient Cas9 expression for 72 h (Plasmids-CaPO_4_)	Germline-Uncut (66.6%)		Editing efficiency (38.4%)	SuperQ
		Indels (35.7%)	Total small deletions (24.0%) Total small insertions (11.3%)		
		Translocations (2.7%)	Between the target site and GW low-level DSB (1.6%) Between the target site and off-target site (1.1%)		
Liu, M. et al. [36]	In vitro CH12F3 (c-myc locus). Transient Cas9 expression for 72 h (Plasmid-nucleofection)	Deletions (69.1%)	≤100 bp (94.3%) 100 bp–3 kb (4.9%) 3–500 kb (0.8%)		PEM-Q
		Insertions (28.0%)	<20 bp (96.1%) ≥20 bp (3.9%)		
		Translocations (2.9%)	Enrichment of translocations at Chr 15 after *c-myc* locus editing		
Hwang, G.H. et al. [37]	In vitro HeLa cells (*PYK2* gene) Transient Cas9 expression for 72 h (Plasmid-Lipofectamine)	Deletions (69.2%) Insertions (13.7%) WT length (17.1%)			CRISPR-sub
	Substitution analysis from 50 endogenous sites in HeLa cells	Substitutions were observed	All sequences (0.80 ± 0.1.52%) WT-length (2.64 ± 3.88%)		
Pallaseni, A. et al. [38]	In vitro Cas9-expressing mESC cells. Transduction with sgRNA-expressing lentivirus	Deletions and Insertions were observed	Large 10 bp+ MH deletions (27%) Small 3–9 bp MH deletions (21%) 1–2 bp deletions (13%) 1–2 bp insertions (15%)	Observed in-frame mutations	FORECasT
Kosicki, M. et al. [39]	In vitro PiggyBac transposons expressing gRNAs introduced into CBA9 Cas9+ cells	Varying indel classes (Deletions and Insertions) of 3 gRNA with different target sequences	gRNA#15: 1 bp insertions, gRNA#48: 1–5 bp deletions gRNA#48: 2, 10, 20 bp deletions	Frame disrupting, frame-preserving (<30 bp insertion or deletion) and in-frame deletions ≥30 bp were discussed	CRISPResso2
Bloh, K. et al. [40]	In vitro H1703 cells with a CRISPR/-Cas9 RNP targeting NRF2 (nucleofection)	Single-gRNA indels (Deletions and insertions observed)	Uncut (0 bp-44.3%) 2 bp deletion (27.6%) 13 bp deletion (10%) 6 bp deletion (5.3%) 3 bp deletion (3.7%) 1 bp insertion (3.3%)	Non-frameshift mutation (53.2%) Frameshift mutation (46.8%)	DECODR
	In vitro LNCAP cells with two gRNAs (RNPs) targeting Rb1(nucleofection)	Dual-gRNA indels (Deletions and Deletion/Insertion indels spanning guides observed)	Guide RNA cut site spanning deletion—61 bp (80.5%) Guide RNA cut site spanning deletions and 1 bp insertion—60 bp (19.5%)	Non-frameshift mutation (19.5%) Frameshift mutation (80.5%)	
Hoellerbauer, P. et al. [41]	In vitro sgRNAs RNPs targeting TP53 or NF1 in GSC and NSC for 24–72 h (nucleofection)	Single gRNA	High frequency of 1 bp insertion and deletion	KO efficiency closely correlated with indel efficiency	ICE
	In vitro two sgRNAs RNPs targeting TP53 or NF1 in GSC and NSC (nucleofection)	Dual gRNA	Guide RNA cut site— spanning deletion—61 bp (53–85%) Guide RNA cut site—spanning deletion ±2 bp (81–93%)		
	In vitro GSC-0827 cells with 3 gRNA RNPs targeting 13 different genomic regions for 5 days	3 gRNA	Deletion spanning the gRNA cut site 1 and 3 Deletions spanning gRNA cut site 1 to 2 or gRNA cut site 2 to 3 No deletion (WT) or small deletions		PCR (No tool stated)
Xiang, X. et al. [42]	In vitro Transient expression of Cas9 and guide gRNAs-Plasmids (4 days post-PEI transfection	Dual-gRNA indels	Deletions spanning gRNA cut sites (45–70%) Deletions spanning gRNA cut sites + insertions or deletions were also observed (NHBEJ)	Dual-guide RNA induced in-frame mutations to restore the reading frame in the *DMD* gene	Deep seq/ICE
Binda, C.S. et al. [43]	In vitro Cas9/gRNAs expressing SupT1 infected with HIV Lai (both gRNAs targeted HIV-1 Gag)	Wild-type (WT) and sequences with mutations, excisions, and inversions were observed	WT or perfect repair (21%) Mutations–insertions or deletions (58%) Excision (21%)	Indicated that an in-frame special mutation may promote viral escape	Sequencing of PCR products (No tool stated)
	In vitro Cas9/gRNAs expressing SupT1 infected with HIV Lai (gRNAs targeted Gag and Tat/Rev)		WT or perfect repair (10%) Mutations–insertions or deletions (44%) Excision (39%) Inversion (7%)		
Wang, G. et al. [44]	In vitro Cas9/dgRNAs expressing SupT1 infected with HIV Lai (Proviral DNA isolated at 12 days post-infection)	WT and sequences with deletions, insertions, and substitutions were observed	WT (25.3%) Deletions (42.9%) Insertions (23.1%) 1–2 nt Substitution (8.8%)	Observation of nucleotide substitutions and 3 nt in-frame insertions in protein-coding sequences of breakthrough viruses. Observed mostly out-of-frame mutations at day 110 post-infection in highly conserved protein-coding sequences	Sequencing of PCR products (No tool stated)
	In vitro Cas9/dgRNAs expressing SupT1 infected with HIV Lai (Proviral DNA isolated 110 days post-infection)		WT (1.9%) Deletions (61.5%) Insertions (32.1%) 1–2 nt Substitution (1.3%) ≥3 nt Substitution (3.2%)		

**Table 2:** CRISPR/Cas9-generated editing profile and outcome prediction tools. This table identifies tools utilized or in part contain a component that can be utilized for the prediction of CRISPR/Cas9 editing, profiles, and outcomes. Term definitions used in the table are as follows: **Year:** Month and year of publication or indicated year submitted on bioRxiv; **Cite:** Citation for tool publication; **Link: Web:** Link to website, web interface, or web browser tool indicated by author (accessed on 2 July 2025, prior to the revised manuscript date); **Link: Command Line Tool:** Link to open-source tool; **# Of Citations:** The number of times the referenced publication has been cited as of August 2025 as per PubMed ID (PMID) Cite [108,109]; **Maintenance:** Reviewed July 2025—Last update indicated on web browser and last commit noted on command line tool; N/A = not indicated or unable to determine; **Model:** Type of model tool was based on; **Single Mode:** Checkmark indicates tool provides option to input or process one sample or gRNA target at a time; **Batch Mode:** Checkmark indicates tool provides option to input or process multiple samples or targets; “-” indicates that it is not provided or not clearly indicated; **Export File Option:** Checkmark indicates tool exports results file from web interface and/or web tool; “-” indicates that it is not provided or not clearly indicated; **Break Type:** Type of break tool focuses on; **Nuclease:** Indicates the nuclease of the PAM that the tool can recognize; **Cell Type:** Cell type tool was trained on or given as an option during tool utilization; **Reference Genome**: Genome gRNAs were based on; **gRNA Length:** Indicates gRNA input length or length of content sequence if gRNA input length not specified. The following predictions are indicated or suggested based on the tool or the literature—**Repair Edit Predicted**: **Insertions:** Checkmark indicates that tool predicts insertions; **Repair Edit Predicted**: **Deletions:** Checkmark indicates that tool predicts deletions; **Repair Edit Predicted: Substitutions:** “-” indicates that substitutions are not predicted or not a primary reported feature; **Repair Edit Predicted**: **Translocations:** “-” indicates that translocations are not predicted; **Repair Edit Predicted: Inversions:** “-” indicates that inversions are not predicted; **Repair Outcome Predicted:** Frameshift: Checkmark indicates that tool predicts direct frameshift frequency, or indirect likelihood of frameshift outcome. Background color identifies sections in table as green: containing tool reference information, peach: indicating tool configuration, light yellow: indicating tool gene editing predictions.

	Tool Features	Apindel	CROTON (CRISPR Outcomes Through cONvolutional Neural Networks)	SPROUT (CRISPR Repair Outcome)	Lindel (Logistic Regression Model to Predict Insertions and Deletions)	inDelphi	FORECasT (Favored Outcomes of Repair Events at Cas9 Targets)
**Reference Information**	**Year**	06/2022	07/2021	09/2019	06/2019	11/2018	11/2018
	**Cite**	[110]	[111]	[31]	[32]	[34]	[30]
	**Link: Web**	N/A	https://github.com/vli31/CROTON	https://zou-group.github.io/SPROUT	https://lindel.gs.washington.edu/Lindel/	https://indelphi.giffordlab.mit.edu	https://elixir.ut.ee/forecast/
	**Link: Command Line Tool**	https://github.com/MoonLBH/Apindel	https://github.com/vli31/CROTON	https://github.com/amirmohan/SPROUT	https://github.com/shendurelab/Lindel	https://github.com/maxwshen/inDelphi-model	https://github.com/felicityallen/SelfTarget
	**# Of Citations**	12	27	87	131	393	342
**Configuration**	**Maintenance**	Last update on command tool 2022.	Last update on command tool 2021.	Last update on command tool 2021. Web browser tool does not work.	Last update on command tool 2019. Web browser page does not work.	Last update on command tool 2018.	Last update on command tool 2021.
	**Model**	GloVe model, Bidirectional Long Short-Term Memory (BiLSTM) network, Attention mechanism, Positional Encoding	Convolutional Neural Network (CNN) and Neural Architecture Search (NAS)	Gradient boosted tree, decision trees	Logistic regression model	Neural network and multitask framework learning, ak-nearest neighbor model	Multi-class logistic regression model
	**Single Mode**	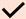	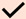	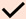	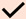	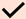	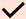
	**Batch Mode**	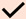	-	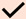	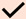	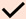	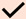
	**Export File Option**	-	-	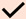	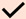	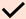	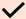
**Gene Editing**	**Break Type**	dsDNA	dsDNA	dsDNA	dsDNA	dsDNA	dsDNA
	**Nuclease**	Cas9	Cas9	SpCas9	SpCas9	SpCas9, KKH SaCas9	SpCas9
	**Cell Type**	K562	Synthetic	Human CD4+ T cells	HEK293T	mESC, U2OS, HEK293, HCT116, K562	K562, CHO, mESC, hiPSC, HAP1, RPE1
	**Reference Genome**	Synthetic, hg19, hg38	Synthetic, hg38	hg38	hg19, Synthetic	hg38, mm10	Synthetic
	**gRNA Length**	60 nt	60 nt	20 nt	20 nt	60 nt	60 nt
	**Repair Edit Predicted: Insertions**	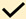	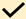	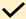	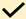	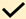	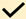
	**Repair Edit Predicted: Deletions**	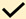	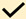	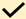	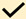	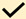	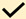
	**Repair Edit Predicted: Substitutions**	-	-	-	-	-	-
	**Repair Edit Predicted: Translocations**	-	-	-	-	-	-
	**Repair Edit Predicted: Inversions**	-	-	-	-	-	-
	**Repair Outcome Predicted: Frameshift**	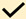	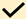	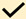	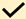	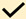	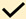

**Table 3:** CRISPR/Cas9-generated editing profile and outcome analytical tools. This table identifies tools utilized or in part contain a component that can be utilized for or primarily supports the analysis of NGS data produced for CRISPR/Cas9 editing, profiles, and outcomes. Term definitions used in the table are as follows: **Year:** Month and year of publication or year submitted on bioRxiv; Cite: Citation for tool publication; **Link: Web:** Link to website, web interface, or web browser tool as indicated by author (accessed on 7 July 2025, prior to the revised manuscript date); N/A = not indicated or not available; **Link: Command Line/Offline Tool:** Link to open-source tool; N/A = not indicated or not available; **# Of Citations:** The number of times the referenced publication has been cited as of August 2025 as per PubMed ID (PMID) Cite [108,109]; **Maintenance:** Reviewed July 2025—Last update indicated on web browser and last commit noted on command line/offline tool; N/A = not indicated or unable to determine; **Documentation Level:** Level of documentation, including environment indicated and instructions for tool use; Basic = minimal instructions and documentation provided; Intermediate = somewhat detailed instructions and documentation provided; Detailed = extensive instructions and documentation provided; **Molecular Prep Technique:** Describes the method or technology used to prepare DNA for downstream analysis by tool; NGS = next-generation sequencing; **Approach:** General method or underlying analytical approach utilized by tool; **Single Mode:** Checkmark indicates tool provides option to input or process one sample or gRNA target at a time; **Batch Mode:** Checkmark indicates tool provides option to input or process multiple samples or targets; **Experimental File Intake Format:** Indicates the experimental file format that the tool supports as input; **Nuclease:** Indicates the Cas nuclease associated to the PAM that the tool can recognize but is not limited to. The following repair edits and outcomes are indicated or suggested based on the tool page, documentation, or literature—**Repair Edit: Insertions:** Checkmark indicates that tool identifies insertions; **Repair Edit: Deletions:** Checkmark indicates that tool identifies deletions; **Repair Edit: Substitutions:** Checkmark indicates that tool quantifies or reports substitutions (substitutions may be implicitly identified from tool output or aligned reads); “-” indicates that substitutions are not identified or not a primary reported feature; **Repair Edit: Translocations:** Checkmark indicates that tool identifies translocations; “-” indicates that translocations are not identified; **Repair Edit: Inversions:** Checkmark indicates that tool identifies inversions: “-” indicates that inversions are not identified; **Repair Outcome: Frameshift:** Checkmark indicates that tool identifies direct frameshift frequency, or indirect likelihood of frameshift outcome, “-” indicates that it is not provided or not clearly indicated by tool literature or webpage. Background color identifies sections in table as green: containing tool reference information, peach: indicating tool configuration, light yellow: indicating tool gene editing details.

	Tool Features	RIMA v2 (Rational InDel Meta-Analysis)	PEM-Q	CRISPAltRations	CRISPR-Sub	ampliCan	SuperQ	CRISPResso2	RIMA v1 (Rational InDel Meta-Analysis)
**Reference Information**	**Year**	08/2023	09/2021	06/2021	06/2020	02/2019	03/2019	03/2019	09/2018
	**Cite**	[144]	[36]	[51]	[37]	[139]	[35]	[141]	[29]
	**Link: Web**	N/A	N/A	https://www.idtdna.com/pages/tools/rhampseq-crispr-analysis-tool	http://www.rgenome.net/crispr-sub/#!	N/A	N/A	https://crispresso2.pinellolab.org/submission	N/A
	**Link: Command Line/Offline Tool**	https://github.com/Ghahfarokhi/ATG_CRISPResso2_to_RIMA2	https://github.com/liumz93/PEM-Q/blob/master	https://idtcrispr.bluebee.com/idtcrispr/	https://github.com/Gue-ho/CRISPR-Sub	Bioconductor Package: http://bioconductor.org/packages/amplican; GitHub: https://github.com/valenlab/amplican	https://github.com/liumz93/superQ	https://github.com/pinellolab/CRISPResso2	https://github.com/Ghahfarokhi/RIMA
	**# Of Citations**	52	64	15	12	45	68	1019	71
**Configuration**	**Maintenance**	Last update on command tool 2024.	Last update on command tool 2024.	Web update 2025.	Last update on command tool 2020. Web update: no maintained date, appears to work.	Last update on command tool 2025.	Last update on command tool 2019.	Last update on command tool 2025. Web update: no maintained date, appears to work.	Last update on command tool 2024.
	**Documentation Level**	Detailed	Intermediate	Detailed	Detailed for web. Basic for offline.	Detailed	Intermediate	Detailed for web and command line.	Detailed
	**Molecular Prep Technique**	NGS	PEM-seq, NGS	NGS	NGS	NGS	PEM-seq, NGS	NGS	NGS
	**Approach**	Variant collection and pattern analysis	Bioinformatic pipeline	Alignment-based pipeline	Statistical comparison	Alignment-based pipeline	Bioinformatic pipeline, deconvolution	Software pipeline, variant calling and alignment	Variant collection and pattern analysis
	**Single Mode**	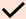	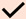	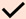	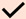	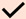	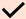	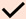	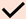
	**Batch Mode**	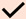	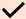	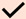	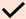	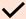	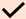	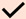	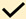
	**Experimental File Intake Format**	Variant Table	FASTQ	FASTQ	FASTQ	FASTQ	FASTQ	FASTQ	Variant Table
**Gene Editing**	**Nuclease**	Cas9	Cas9 family, Cas12a/e/f/j family	Cas9, Cas12a, Contact for other nucleases	SpCas9, SpCas9-VQR, SpCas9-EQR, SpCas9-VRER, SaCas9, SaCas9-KKH, Spy-macCas9, XCas9 3.7	Cas9	Cas9	SpCas9, Cpf1, None	Cas9
	**Repair Edit: Insertions**	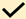	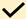	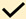	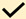	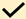	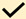	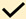	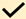
	**Repair Edit: Deletions**	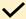	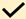	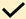	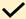	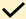	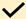	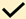	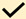
	**Repair Edit: Substitutions**	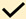	-	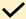	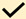	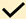	-	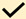	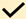
	**Repair Edit: Translocations**	-	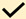	-	-	-	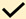	-	-
	**Repair Edit: Inversions**	-	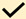	-	-	-	-	-	-
	**Repair Outcome: Frameshift**	-	-	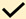	-	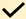	-	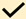	-

**Table 4:** CRISPR/Cas9-generated editing profile and outcome analytical tools. This table identifies tools utilized or in part contain a component that can be utilized or primarily supports the analysis of data produced from Sanger sequencing, identifying CRISPR/Cas editing, profiles, and outcomes. Term definitions used in the table are as follows: **Year:** Month and year of publication or year submitted on bioRxiv; Cite: Citation for tool publication; **Link: Web:** Link to website, web interface, or web browser tool as indicated by author (accessed on 7 July 2025, prior to the revised manuscript date); N/A = not indicated or not available; **Link: Command Line/Offline Tool:** Link to open-source tool; N/A = not indicated or not available; **# Of Citations:** The number of times the referenced publication has been cited as of August 2025 as per PubMed ID (PMID) Cite [108,109]; **Maintenance:** Reviewed July 2025—Last update indicated on web browser and last commit noted on command line/offline tool; N/A = not indicated or unable to determine; **Documentation Level:** Level of documentation, including environment indicated and instructions for tool use; Basic = minimal instructions and documentation provided; Intermediate = somewhat detailed instructions and documentation provided; Detailed = extensive instructions and documentation provided; **Molecular Prep Technique:** Describes the method or technology used to prepare DNA for downstream analysis by tool; NGS = next-generation sequencing; **Approach:** General method or underlying analytical approach utilized by tool; **Single Mode:** Checkmark indicates tool provides option to input or process one sample or gRNA target at a time; **Batch Mode**: Checkmark indicates tool provides option to input or process multiple samples or targets; “-” indicates that it is not provided or not clearly indicated; **Experimental File Intake Format:** Indicates the experimental file format that the tool supports as input; **Nuclease:** Indicates the Cas nuclease associated to the PAM that the tool can recognize but is not limited to. The following repair edits and outcomes are indicated or suggested based on the tool page, documentation, or literature—**Repair Edit: Insertions:** Checkmark indicates that tool identifies insertions; **Repair Edit: Deletions:** Checkmark indicates that tool identifies deletions; **Repair Edit: Substitutions:** Checkmark indicates that tool quantifies or reports substitutions (substitutions may be implicitly identified from tool output or aligned reads); “-” indicates that substitutions are not identified or not a primary reported feature; **Repair Edit: Translocations:** “-” indicates that translocations are not identified; **Repair Edit: Inversions:** “-” indicates that inversions are not identified; **Repair Outcome: Frameshift:** Checkmark indicates that tool identifies direct frameshift frequency, or indirect likelihood of frameshift outcome, “-” indicates that it is not provided or not clearly indicated by tool literature or webpage. Background color identifies sections in table as green: containing tool reference information, peach: indicating tool configuration, light yellow: indicating tool gene editing details.

	Tool Features	ICE (Inference of CRISPR Edits)	DECODR (Deconvolution of Complex DNA Repair)	CrispRVariants	CRISP-ID	TIDE (Tracking of Indels by DEcomposition)
**Reference Information**	**Year**	02/2022	11/2021	07/2016	07/2016	09/2014
	**Cite**	[143]	[40]	[142]	[140]	[145]
	**Link: Web**	https://ice.editco.bio/#/	https://decodr.org/	http://imlspenticton.uzh.ch:3838/	http://crispid.gbiomed.kuleuven.be	https://tide.nki.nl/
	**Link: Command Line/Offline Tool**	https://github.com/synthego-open/ice	N/A	CRISPRVariants: http://bioconductor.org/packages/CrispRVariants; CRISPRVariantsLite: https://github.com/markrobinsonuzh/CrispRvariantsLite	N/A	R code available upon request
	**# Of Citations**	532	76	112	167	1700
**Configuration**	**Maintenance**	Last update on command tool 2019. Web update 2025.	N/A	Last update on command tool manual 2025. Web update—access may be unavailable or restricted.	Web update 2025—moved to local only.	Web update—no maintained date appears to work.
	**Documentation Level**	Detailed	Detailed	Detailed	Detailed	Detailed
	**Molecular Prep Technique**	Sanger Sequencing	Sanger Sequencing	Sanger Sequencing, High-Throughput Sequencing	Sanger Sequencing	Sanger Sequencing
	**Approach**	Linear regression	Proposal generation and determination algorithm	R toolkit, variant clustering and alignment	Trace deconvolution algorithm	Decomposition algorithm
	**Single Mode**	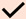	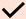	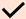	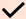	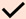
	**Batch Mode**	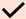	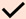	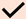	-	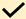
	**Experimental File Intake Format**	AB1	AB1, FASTA	AB1, FASTQ derived, BAM	AB1, SCF	AB1, SCF
**Gene Editing**	**Nuclease**	SpCas9, Cas12a, hfCas12Max, MAD7, eSpOT-ON, None	SpCas9, Cas12a, None	Cas9	Cas9	Cas9, Other RNA guided nucleases
	**Repair Edit: Insertions**	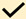	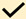	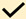	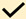	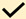
	**Repair Edit: Deletions**	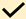	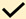	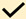	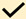	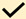
	**Repair Edit: Substitutions**	-	-	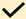	-	-
	**Repair Edit: Translocations**	-	-	-	-	-
	**Repair Edit: Inversions**	-	-	-	-	-
	**Repair Outcome: Frameshift**	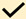	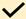	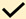	-	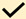

## Data Availability

No new data were created or analyzed in this study. Data sharing is not applicable to this article.
